# A Protocol Design Paradigm for Batched Sparse Codes

**DOI:** 10.3390/e22070790

**Published:** 2020-07-20

**Authors:** Hoover H. F. Yin, Raymond W. Yeung, Shenghao Yang

**Affiliations:** 1 Institute of Network Coding, The Chinese University of Hong Kong, Shatin, New Territories, Hong Kong; 2 Department of Information Engineering, The Chinese University of Hong Kong, Shatin, New Territories, Hong Kong; 3 School of Science and Engineering, The Chinese University of Hong Kong, Shenzhen, Shenzhen 518172, China; shyang@cuhk.edu.cn; 4 Shenzhen Key Laboratory of IoT Intelligent Systems and Wireless Network Technology, The Chinese University of Hong Kong, Shenzhen, Shenzhen 518172, China; 5 Shenzhen Research Institute of Big Data, Shenzhen 518172, China

**Keywords:** batched network coding, BATS codes, protocol, recoding, burst packet loss, interleaving

## Abstract

Internet of Things (IoT) connects billions of everyday objects to the Internet. The mobility of devices can be facilitated by means of employing multiple wireless links. However, packet loss is a common phenomenon in wireless communications, where the traditional forwarding strategy undergoes severe performance issues in a multi-hop wireless network. One solution is to apply batched sparse (BATS) codes. A fundamental difference from the traditional strategy is that BATS codes require the intermediate network nodes to perform recoding, which generates recoded packets by network coding operations. Literature showed that advanced recoding schemes and burst packet loss can enhance and diminish the performance of BATS codes respectively. However, the existing protocols for BATS codes cannot handle both of them at the same time. In this paper, we propose a paradigm of protocol design for BATS codes. Our design can be applied in different layers of the network stack and it is compatible to the existing network infrastructures. The modular nature of the protocol can support different recoding techniques and different ways to handle burst packet loss. We also give some examples to demonstrate how to use the protocol.

## 1. Introduction

The Internet is no more only accessed by computers or smartphones. Billions of everyday objects like smart home devices constituent the Internet of Things (IoT). Wireless networks, including mesh and ad-hoc networks, have the important role of connecting the large number of IoT devices. However, the traditional network protocols designed for the Internet, for example, Transmission Control Protocol (TCP), could have poor performance for general wireless networks. To realize the full potential of IoT, it is necessary to design a more efficient network protocol that is suitable for most practical environments. The development of network coding [1,2] over the past decade have resulted in feasible network codes for this purpose, for example, batched sparse (BATS) codes [3,4,5].

### 1.1. Issues of Traditional Approaches for Wireless Networks

In traditional wireless networks, for example, WiFi networks, devices are connected to the wired Internet backbone within one wireless hop. Seldom do we see a traditional wireless network having multiple wireless hops unless it is for specific purposes like deep-space [6] and deep-sea communications [7]. In contrast, the evolution of IoT diversifies the wireless network topologies [8,9]. The mobility of devices is achieved by means of employing multiple wireless links [10].

One example is the vehicular ad hoc network (VANET) [11], where the vehicle-to-vehicle and vehicle-to-infrastructure communications pass data through multiple potentially moving vehicles and road side units (RSUs) via wireless links. On the other hand, an RSU usually is a static object on the road side, for example, a lamppost. However, it is not always feasible to have a wired connection from an RSU to the Internet or other networks due to the substantial installation work and cost for optical fibers or Ethernet cables. In other words, the transmitted data may need to go through multiple wireless links between RSUs before it can reach a wired network.

Another example is to provide Internet access to rural and remote areas [12]. In some cases, even cellular signals cannot reach certain locations due to the mask of terrain. It is not cost-efficient to cover the whole area with wired Internet access, so installing multi-hop wireless relay networks is a potential direction, for example, Project Loon (https://loon.com/), which uses high-altitude balloons as relays.

A major challenge in deploying multi-hop wireless networks is that it is a common phenomenon to have packet loss in wireless communications [13]. In a wired network, packet loss is mainly due to congestion. However in a wireless network, it may also be due to signal fading, interference, and so forth. When the forward error correction code, if exists, cannot recover the received data, then the data is considered corrupted. Link-by-link retransmission scheme exists in some standard like IEEE 802.11 which can increase the chance of providing correctly received data to the upper layers of the network stack. Error detection is further performed in different layers, for example, the cyclic redundancy check code of medium access control (MAC) in the data link layer, and the Internet checksum of Internet Protocol (IP) and Transmission Control Protocol (TCP) in the network layer and transport layer respectively. Once an error is detected, the whole piece of data is dropped. We call the lost data the lost packets. Although for different layers, we should use the corresponding units frame, packet, segment, and so forth, we will only use the unit *packet* for simplicity.

In the traditional network setting, the intermediate network nodes, for example, routers and switches, adopt the store-and-forward strategy. We simply call a network node a *node* when it is clear from the context. Once a packet is lost in one of the links, no matter it is wired or wireless, the packet cannot reach the destination node. Note that not all applications require the destination node to receive all the packets, for example, live streaming. But when a reliable transmission is needed, for example, downloading files, it is usually the role of transport layer protocols to provide such a service. The current Internet mostly depends on TCP for the reliability.

TCP adopts an end-to-end feedback mechanism at the cost of degraded system performance, where the performance degradation is mainly due to the delay and the bandwidth consumption of feedback. When a packet is lost, an end-to-end retransmission of that packet is triggered and the rate control algorithm of TCP reduces the transmission rate. However, TCP was originally designed for wired network, where packet loss is mainly due to congestion. Reducing the transmission rate cannot handle other causes of packet loss in wireless communications. Variations of TCP like ad hoc TCP (ATCP) [14] were proposed to handle these kinds of packet loss events. These TCP-alike protocols still rely on feedback. In some extreme applications like deep-space and deep-sea communications, feedback has a very long delay or may not even be available. On the other hand, although an end-to-end retransmission scheme works well in networks with only a few wireless links, it is not the case when the number of wireless links becomes large. It is because a packet has a higher chance to be lost at one of the links. This also increases the number of retransmissions so that the system performance is further degraded. Such performance degradation happens in end-to-end retransmission schemes including TCP-alike protocols. For example, Fast Adaptive and Secure Protocol (FASP) [15], which is also known as Aspera, only sends feedback to ask for retransmission when a packet is lost, but the retransmitted packet still has a high chance to be lost at one of the wireless links when the number of wireless links is large.

In a traditional application layer protocol, the data to be transmitted is encapsulated by the lower layers directly without applying any redundancy. When a packet is lost, we need to retransmit the packet as the data in the packet does not appear in any other packet. In other words, if we want to get rid of feedback or retransmission, then we need to encode the data so that the encoded packets are mixtures of different parts of the data. Upon knowing that the fundamental design of TCP is not suitable for the IoT era, approaches that do not rely on either feedback or retransmission were developed, for example, fountain codes [16], random linear network codes (RLNC) [17,18,19,20,21], and so forth.

*Fountain codes*, for example, Luby transform (LT) codes [16], Raptor codes [22,23] and online codes [24], can generate potentially unlimited number of packets from the input data, which is known as the *ratelessness* property. The destination node can recover the data by belief propagation (BP) decoding after enough packets are received. There is no requirement on the sequential order of the received packets. Furthermore, the performance of a fountain code is not affected by the packet loss pattern. However, the chance of receiving a packet successfully can be low when the number of wireless links is large. In this case, the source node has to transmit an enormous number of packets, which is clearly inefficient. The performance of fountain codes is no better than an end-to-end retransmission scheme which has ideal (instantaneous, reliable and bandwidth cost free) feedback.

### 1.2. Network Coding Based Approaches

*Network coding* [1,2] in general has throughput gain over forwarding by allowing the intermediate network nodes to transmit new packets generated by the received packets. *Random linear network coding (RLNC)* is a distributed realization of network coding which can achieve the capacity of networks with packet loss for a wide range of scenarios [25,26,27]. The input data is divided into multiple *input packets*, where each packet is regarded as a vector in a finite field. The source node generates and transmits random linear combinations of the input packets, that is, the coefficients of the linear combinations are randomly chosen from the underlying field of the vector space. Instead of forwarding, the intermediate nodes generate packets by taking random linear combinations of the already received packets. The destination node recovers the data by solving the corresponding system of linear equations by Gaussian elimination. In order to know how a packet is formed by the random linear combination, a coefficient vector is prepended to the payload. The overhead induced by the coefficient vector can be significant when there are many input packets. Also, the intermediate nodes have high storage and computational costs. However, the intermediate nodes are usually routers or embedded devices having small storage and low computational power, which inhibits the deployment of RLNC.

The concept of *batched network coding (BNC)*, which is also known as segmented network coding or chunked network coding, was proposed in Reference [19]. BNC is a practical realization of RLNC which overcomes the disadvantages of RLNC. The core idea is to encode the input packets into small batches of coded packets and restrict the RLNC operations on the coded packets belonging to the same batch. Depending on the code, a batch is also known as a generation [19], a chunk [28], a class [29], a segment [30], and so forth. To generate a batch, we can use disjoint subsets of the input packets [19,31], overlapped subsets of the input packets [28,29,32,33], or even small subsets of the coded packets generated from the input packets [3,4,34,35,36]. The input packets for generating batches may include coded packets generated from the original input packets. For convenience, we refer to those coded packets generated from the input packets before applying a batched network code simply as the input packets. As a batch only contains a small number of encoded packets, the coefficient vectors overhead and the computational cost at the intermediate nodes are small. The intermediate nodes can discard a batch after the RLNC operations are done and the packets in the batch are transmitted, so the storage cost is also small.

By using disjoint subsets of input packets to form generations (batches) of BNC (called *disjoint BNC*), the applications of RLNC has been extensively studied and experimented [37,38,39,40]. The fundamental complexity and overhead issues of RLNC limit the generation size to a small number, for example, 16 or 32. Existing protocols using disjoint BNC [38,39,40] must employ complicated feedback mechanisms to assist the coded packet generation for coding efficiency, so that they are not suitable for networks with links having long delay and low reliability. For a file with a large number of packets, many disjoint batches must be handled independently, and hence these approaches face essentially the same problem as transmitting each packets individually. To resolve this problem of disjoint BNC, overlapped and coded batches should be applied, where no feedback is required for the reliable transmission of each individual batches. Note that we do not exclude the possibility of using feedback for collecting the network status information, which may help to improve the communication throughput. For example, when the packet loss rate is fixed and known on a network link, it is not necessary to have further feedback to guarantee the reliability and throughput of coded BNC, while feedback must be employed link-by-link for disjoint BNC to guarantee the reliability and throughput.

*Batched sparse (BATS) code* [3,4,5] is a batched network code that can asymptotically achieve rates very close to the capacity of a packet network with packet loss, and at the same time can be implemented in most practical settings. A BATS code consists of an *outer code* and an *inner code*. The outer code is a matrix generalization of fountain code that preserves the ratelessness property of fountain codes, that is, a potentially unlimited number of batches can be generated. Also, the data can be recovered after receiving enough batches. The inner code performs network coding operations on the packets belonging to the same batch, where these operations are also called re-encoding or simply *recoding*. There are techniques other than RLNC for generating recoded packets, for example, systematic recoding [5,41]. It is known that the design of the inner code has impact on the achievable rates [41], for example, the advanced recoding schemes in References [30,42,43,44]. On the other hand, Reference [41] also suggested that burst packet loss can reduce the advantage of using BATS codes. A broad range of application scenarios of BATS codes have been discussed in the literature, including wireless ad-hoc networks [45], underwater acoustic networks [45,46], deep space networks [47] and edge/fog computing [48,49].

As a summary of the approaches described above, we list some protocol independent properties in Table 1. A similar table can also be found in Reference [5]. We consider an *ℓ*-hop line network where each link has a packet loss rate $p_{E}$. The capacity of the network is $1-p_{E}$. In the table, *K* is the number of input packets, *T* is the length of a packet, and *M* is the number of coded packets in a batch where *M* is usually much smaller than *K*.

As discussed above, we are looking for an approach which does not require feedback for reliability. Among the first three approaches in Table 1, RLNC has the highest asymptotic throughput but it also has the highest storage cost and highest coding coefficient overhead, so it is only suitable for the scenarios when *K* is small. BATS codes have an asymptotic throughput close to that of RLNC, but the storage cost and the coding coefficient overhead are much smaller than that of RLNC when *K* is large. However, BATS codes suffer from performance degradation in case of burst loss, but it can be alleviated by applying interleavers that spread the burst. The drawback is that the storage cost is multiplied by a factor of O(L) where *L* is the depth of the interleaver. As the interleaver depth is not very large in general, the increased storage cost is still acceptable. Therefore, BATS codes are suitable for multi-hop wireless networks where feedback is not ideal.

To adopt BATS codes in practice, we need to use a *BATS code based network transmission protocol*, simply called a *BATS protocol*. A simple prototype of a BATS protocol named BATS-pro1 was proposed in Reference [41]. Features like out-of-order packets handling and timeout for packets in the buffer are not included in the prototype. In BATS-pro1, batches are transmitted sequentially. When a *baseline recoding scheme* which generates the same number of packets for every batch is applied, burst loss can easily be handled by a block interleaver. However, when advanced recoding schemes like *adaptive recoding* [30,44] are applied, a different number of packets are generated for different batches, so that application of block interleavers becomes more difficult. Another protocol called *joint fountain coding and network codes (FUN codes)* [50,51] captures the two-way transmission session of BATS codes. Its implementation injects an extra layer to the network stack, which restricts the use of FUN codes in a closed network as every node has to implement the handling of the extra layer.

### 1.3. Our Contributions

In order to realize the full potential of BATS codes in practical systems, we need a framework of a BATS protocol which is designed for enhancing and preserving the advantage of BATS codes. In this paper, we propose a paradigm of designing BATS protocols that mainly includes the following components/mechanisms:

- The packet structure that includes the necessary information passing to support BATS code recoding and decoding.
- The BATS protocol modules at the source, intermediate and destination nodes for BATS code encoding, recoding and decoding.

The paradigm focuses on the operations of a single transmission session of BATS codes. When there are multiple sessions in the network, we may need to consider fairness among the sessions [52,53] and transmission schedules that can avoid collisions [54], which can be implemented by another layer of high-level protocol around a BATS protocol. The details of such an implementation are beyond the scope of this paper.

The paradigm of designing BATS protocols provides an abstract encapsulation of the BATS code operations so that the details of BATS code operations are transparent to a high-level protocol. The BATS protocol modules allow a general class of encoding, recoding and decoding operations. In particular, the paradigm can

- use arbitrary recoding schemes like the advanced adaptive recoding [30,44] to improve the throughput; and
- use burst loss handling techniques like interleaving to preserve the advantage of BATS codes.

The paradigm also enables the use of BATS protocol in existing network infrastructures. One of the applications of this paradigm is to implement a protocol that can carry other Internet traffic through multiple wireless links in a way which is transparent to the protocols in other layers, that is, the end-users do not need extra software or configuration in order to enjoy the benefits provided by BATS codes.

In addition to the BATS protocol design, we also propose and discuss some extensions on the existing BATS recoding schemes which make BATS codes more suitable to be applied in practical systems. In particular, we discuss

- the reduction of the computational cost at the intermediate nodes by supporting a smaller finite field for recoding;
- the application of causal recoding which generates real-time partially recoded packets; and
- the transformation of a packet loss model to suit the unified framework for adaptive recoding schemes.

### 1.4. Organization of the Paper

The rest of the paper is organized as follows. We first give a brief introduction to BATS codes in Section 2. In Section 3, we describe some existing recoding schemes which can be used by BATS codes. Some mathematical discussions about recoding are deferred to Appendix A, Appendix B and Appendix C. Our proposed module design and packet design of the BATS protocol paradigm are presented in Section 4 and Section 5 respectively. Then, some examples are given in Section 6 to demonstrate how to use the BATS protocol. We conducted some simulations on the BATS protocol in Section 7 using an artificial network to demonstrate the performance of the protocol. Finally, we conclude the paper in Section 8. We summarize the organization of the paper in Table 2.

## 2. BATS Codes

We give an introduction to BATS codes in this section. We refer readers to Reference [5] for a more detailed discussion on the encoding and decoding of BATS codes.

There are three basic components in BATS codes: the encoder, the recoder and the decoder. The encoder and decoder are applied at the source and destination nodes respectively. The recoders, which can be employed at the source node and the intermediate nodes, are the core components of BATS codes applying the network coding operations. Without recoders, we would have a similar drawback as fountain codes in multi-hop wireless networks. Recoding schemes will be discussed separately in Section 3.

We need to use a separate BATS encoder for the input data of each *information source*. Similarly, each BATS decoder corresponds to the input data of one information source. An information source can be a file, a bit string, a network packet, and so forth. We can also partition a file into multiple subfiles and regard each subfile as an information source.

### 2.1. Base Field and Recoding Field

We need a finite field to define the operations involved in a linear combination. It is known that a larger field size can result in a better throughput for BATS codes [4]. This is due to the fact that a newly generated packet by random linear combination has a lower chance to be linearly dependent with the already generated packets at the same node when the field size is larger. Although taking a field size tends to infinity can achieve the best outcome, it is not practical as the symbols in such a field cannot be represented by a finite amount of memory.

The finite field operations can be computationally heavy for the recoders even for a small field like $\mathrm{GF}(2^{8})$. The recoders are applied at the intermediate nodes, which are mostly routers or embedded devices having low computational power. Unless using a specifically designed hardware like in Reference [55], all the operations are required to be done by the general purpose computational units inside the devices. *Single instruction, multiple data (SIMD)*, if supported, can speed up the calculation. A huge range of algorithms for hardware and software for finite field operations have been developed [56,57,58,59]. Precomputed lookup table is another efficient method to reduce real-time computations [58,59,60,61]. In some instruction set architectures, there may also be a SIMD table lookup instruction, for example, the Supplemental Streaming SIMD Extensions 3 (SSSE3) instruction pshufb, which corresponds to the Intel intrinsic _mm_shuffle_epi8.

The computational cost is highly reduced if we use a binary field, as it only requires exclusive-or operations which are natively supported by most central processing units (CPUs). If we measure the actual time used to transmit and decode, it is suggested in Reference [62] that the overall time is shorter if we use a binary field for all the field operations.

In light of this, we consider two different fields in this paper so that the encoder can use a larger field for higher throughput while the recoders can use a smaller field to reduce the computational time. Fix a finite field GF(q) and another finite field $\mathrm{GF}(q^{\prime })$, where *q* is a power of $q^{\prime }$ so that the field $\mathrm{GF}(q^{\prime })$ is a subfield of GF(q). We call them the *base field* and *recoding field* respectively. The base field is used for BATS encoding and decoding, while the recoding field is used for BATS recoding. This generalizes the typical case that recoding uses the same finite field as encoding and decoding. Each symbol in GF(q) is equivalent to an ordered $\left(\log_{q^{\prime }}q\right)$-tuple of symbols in $\mathrm{GF}(q^{\prime })$. In this way, we can use a larger base field like $\mathrm{GF}(2^{8})$ and a much smaller recoding field like the binary field.

### 2.2. Encoding

A packet is regarded as a column vector of length T>0 over GF(q), or equivalently, a column vector of length $T\log_{q^{\prime }}q$ over $\mathrm{GF}(q^{\prime })$. A set of packets is equated to a matrix formed by juxtaposing the packets in this set.

**Definition** **1.** *A totally random matrix is a matrix which has uniform independent and identically distributed (i.i.d.) components.*

We first divide the data to be encoded into K>0 input packets (of length *T* over GF(q)). We can append dummy symbols to extend the length of the last input packet if its length is less than *T*. The encoder applies the *outer code* to the *K* input packets to generate a sequence of *batches*, where each batch is a set of coded packets generated from a subset of the *K* input packets. We call the number of coded packets in a batch the *batch size* of this batch.

We can further apply *precoding* [22,23] before we start the encoding procedure. A precode is actually a traditional erasure code. When the data to be encoded is first processed by a precode, we can stop the decoding once we have recovered a sufficient number of the input packets for the precode to recover the original data.

Similar to fountain codes, a degree distribution is used to for choosing the *degree*, that is, the number of input packets to be selected for the encoding of a batch. The original BATS codes in Reference [3] use a fix batch size for all batches, and it was generalized in Reference [63] that different batches can use different batch sizes, though it is optimal to use the same batch size for all the batches in some scenarios like the one considered in Reference [64]. The distribution we need is a *batch degree and batch size joint distribution* $\Pi =(\Pi_{1,1},\ldots ,\Pi_{d_{\max},m_{\max}})$, where $d_{\max}$ and $m_{\max}$ are the maximum batch degree and the maximum batch size respectively. We also call this distribution the *degree distribution* for the sake of convenience. The distribution can be obtained by solving an optimization problem [63]. When *K* is small, a better distribution can be obtained via the approach in References [65,66,67]. There are also some variants on the degree distribution optimization problem which apply a sliding window [68] or an expanding window [69]. When the distribution is optimized, we can guarantee that a given fraction of the input packets can be recovered with high probability.

All batches are generated independently by the following steps:

1. Sample a batch degree $d_{i}$ and batch size $m_{i}$ from the batch degree and batch size joint distribution Π with probability $\Pi_{d_{i},m_{i}}$.
2. Pick $d_{i}$ packets from all the *K* input packets uniformly and juxtapose them into a matrix $B_{i}$.
3. The *i*-th batch $X_{i}$ is generated by
  $$X_{i}=B_{i}S_{i},$$
  where the batch generator matrix $S_{i}$ is a $d_{i}\times m_{i}$ totally random matrix over the base field.

We can see that the (coded) packets in a batch are formed by taking random linear combinations of the selected input packets.

The batches of coded packets generated by the source node are transmitted to the destination node through the network. At the source node and each intermediate node, the batches are recoded by the *inner code*, where recoding is applied to coded packets belonging to the same batch. Different recoding schemes will be discussed in detail in Section 3.

### 2.3. Decoding

The packets in a batch can be lost when they pass through the network. In any case, since the operations of the inner code are linear, the packets in a batch arriving at the destination node are linear combinations of the packets of the same batch generated by the encoder. Therefore, we can represent the *i*-th batch at the destination node by

(1) $$Y_{i}=X_{i}H_{i}=B_{i}S_{i}H_{i},$$

where $H_{i}$ is an $m_{i}$-row matrix called the *batch transfer matrix*. The number of columns in $H_{i}$ corresponds to the number of packets received for the *i*-th batch, which is finite. Here, $Y_{i}$ and $S_{i}H_{i}$ are over the base field.

We can apply the BP decoding algorithm to decode a BATS code efficiently. The BP decoder for BATS codes with variable batch sizes was analyzed in Reference [63]. There are also some improved BP decoders for BATS codes, for example, Reference [70].

The basic idea of the BP decoding for BATS codes is as follows. The input of a BP decoder is a sequence of ordered pairs $(Y_{i},S_{i}H_{i}),i=1,\ldots ,n$, where *n* is the number of batches transmitted. The batch transfer matrix $H_{i}$ can be obtained from the coefficient vectors. The batch generator matrix $S_{i}$ can be known when the index of the batch, *i*, is identified, which can be achieved by assigning a unique batch ID to each of the packets. If $\mathrm{rk}\left(S_{i}H_{i}\right)=d_{i}$, then the *i*-th batch is said to be *decodable*. The input packets of a decodable batch can be recovered by solving (1), which is a system of linear equations. The recovered input packets are then substituted into the related batches which are not decodable. The BP decoder keeps looking for decodable batches and stops when all batches are decoded or no more batches are decodable.

When the asymptotically optimized degree distribution like the one in Reference [63] is used for a small number of input packets, the BP decoder tends to stop before the desired fraction of input packets are decoded. *Inactivation decoding* techniques [23,71] can be applied to continue the decoding process after the BP decoder stops. One way to perform inactivation decoding is that an undecoded input packet is picked and marked as inactive when no decodable batches are left. Although the inactive packet is an indeterminate, it is substituted into the batches just like a decoded packet. Another way to perform inactivation decoding is to select a small subset of input packets and mark them as inactive at the encoder before the batches are generated. Basically, inactivation decoding trades extra computational cost for a smaller coding overhead.

### 2.4. Ranks of Batches

Although the contents of the packets in a batch can be linearly dependent of each other, the inner code defines another notation for the linear dependency of the packets.

**Definition** **2.** *Two packets are linearly independent if and only if their coefficient vectors are linearly independent.*

In the above definition, the linear dependency of the packets are recorded by their coefficient vectors. These coefficient vectors can be prepended to the packets in practice. We leave the discussion on the design of these initial coefficient vectors to Section 5.2.3.

**Definition** **3.** *The rank of a batch is the number of linearly independent packets in the batch. A rank distribution is the probability distribution of the ranks of the batches.*

The ranks of the batches have an important role in designing the recoding scheme. We can regard the rank of a batch as a measure of the amount of useful information carried by the batch.

The coded packets of a newly generated batch by the encoder are regarded as linearly independent of each other by the inner code. That is, a batch carries the most information when it is newly generated. It is likely that the rank drops after the batch has traversed the network. The expected value of the rank distribution of all the batches at the destination node is the theoretical upper bound on the achievable rate [72], where a BATS code with an optimal degree distribution can achieve rates very close to this upper bound [3]. Therefore, the expected value of the rank distribution at the destination node can be regarded as a close approximation to the throughput of a BATS code.

We define two special terms to simplify the discussions in this paper.

**Definition** **4.** *The input rank distribution at a network node is the rank distribution of the batches arriving at the node.*

**Definition** **5.** *The expected rank is the expected value of the rank distribution of the batches arriving at the next network node.*

Note that in the definition of the expected rank, we consider the next network node in lieu of the current node. The reason is that in adaptive recoding schemes [30,44], the goal is to find the optimal number of recoded packets such that the batches can sustain the most information at the next node.

## 3. Recoding Schemes

The inner code of a BATS code (at the source node or an intermediate node) refers to the network coding operations on the coded packets. Those operations are restricted to coded packets belonging to the same batch unless it is specifically designed not to do so like in FUN codes [50,51]. Some basic questions we have to answer are the following:

1. How to generate the recoded packets?
2. How many recoded packets should be generated?
3. How to transmit the recoded packets?

RLNC is one of the solutions to the first question, that is, the recoded packets of a batch are generated by taking random linear combinations of all the received packets of this batch. *Systematic recoding scheme* [5,41] is another approach, which treats part of the received packets as recoded packets, but it is not feasible in all network topologies, for example, wireless relay networks [73]. In this section, we will further introduce *causal recoding*, which is a more practical recoding approach that can adapt to the dynamics of packet receiving and transmitting rates.

The second question was raised in Reference [41] and has been discussed in Reference [5] for the case that all the batches has the same number of recoded packets. In general, it is not optimal to transmit the same number of recoded packets for every batch. However, Reference [41] did not propose a method to find the optimal solution. Later, designs focusing on the optimal solution which can produce more efficient BATS codes were proposed in References [30,44]. These approaches are referred to as adaptive recoding.

The third question is related to the transmission policy. For example, transmitting batch-by-batch can reduce the storage required at the intermediate nodes because a transmitted batch can be safely discarded. However in a burst loss scenario, the performance of BATS codes would be degraded if we transmit the recoded packets batch-by-batch [41]. It may be due to the fact that some batches lose too many packets. The burst loss effect can be alleviated by transmitting the recoded packets of different batches in an interleaved manner. When there are multiple users in the network, we may need to consider the fairness among the users [52,53] and transmission schedules that can avoid collisions [54].

We need to assume some reasonable behaviours of a recoder before we can propose a mathematical model. Proper recoding [5] is one of the ways to define such an assumption. We leave the discussion on proper recoding to Appendix A.

In this section, we discuss some details regarding the recoding procedure. Before we start, we define some terminologies for simplicity.

**Definition** **6.** *Receivable packets of a batch are the packets of this batch arriving at a network node over all time that are not dropped by the previous link.*

**Definition** **7.** *A useful packet of a batch is a packet which is linearly independent of the already received packets of the same batch at the next network node. A useless packet of a batch is a packet which is not a useful packet of the batch.*

### 3.1. Baseline Recoding

We first describe a fundamental recoding scheme. When BATS codes were first introduced, the number of recoded packets to be generated by the recoding procedure is the same for all batches. This approach is called the *baseline recoding scheme*, or the *opportunistic recoding scheme* in some texts. Note that the baseline recoding scheme is in general not throughput optimal [41], but it is used as a benchmark to compare with other recoding schemes.

The deterministic property, owing to its simplicity in both implementation and analysis of the baseline recoding scheme, has induced many research results, for example, the discrete first-order stochastic dominance [74,75] property of the rank distributions on line networks was investigated in Reference [64], and the batch transfer matrices were shown to be diagonalizable in Reference [43]. Also, the protocol BATS-pro1 [41] and FUN codes [50,51] are also based on the baseline recoding scheme.

When dealing with burst packet loss, a block interleaver [76] can naturally be applied to spread the burst loss over time. The nature of the block interleaver here is different from the traditional one where the intermediate nodes only perform forwarding. We have to receive and store the packets of the batches arriving at a node before we can perform recoding, which means that we need to deinterleave an interleaved block for recoding and then reinterleave it later for transmission. This gives us the freedom to select different interleaver depths at different intermediate nodes to suit the channel conditions of the outgoing links. Some analysis on this kind of block interleaver for baseline recoding schemes can be found in References [77,78].

### 3.2. Adaptive Recoding

Recall that a BATS code can achieve rates very close to the expected value of the rank distribution of all the batches at the destination node. Such a rank distribution is affected by the channel conditions of the network and also the recoding operations by the intermediate nodes. In other words, we can optimize this expected value for a better throughput.

To see why the recoding operations have impact on the expected value of the rank distribution at the destination node, we give a simple but extreme example below. Suppose we receive two batches at an intermediate node, where one of them has rank one and the other one has rank equals to its batch size. If we apply the baseline recoding scheme, the recoded packets of the former batch can be regarded as a repetition code with huge redundancy, because the next node only has to receive one of the recoded packets of this batch in order to recover all the information carried by this batch. For the other batch, we have to receive at least the same amount of recoded packets as its batch size in order to be able to recover all the information carried by this batch. However, the baseline recoding scheme does not generate any redundancy for this batch, so it is likely that some packets from this batch will be lost when we transmit these recoded packets through a lossy link, which incurs information loss. We can intuitively see that it is beneficial to generate and transmit fewer recoded packets for a batch having a lower rank and more recoded packets for a batch having a higher rank. This way, the expected value of the rank distribution at the destination node can potentially become larger.

After Reference [41] had pointed out the suboptimality of the baseline recoding scheme, optimization problems known as *adaptive recoding schemes* were formulated in References [30,44]. Both schemes can adapt fluctuations in the number of packets lost in the individual batches by optimizing the expected rank (at the next node). It is because the local information a node has is the input rank distribution and the channel conditions of the outgoing links. Hence, these schemes can be applied distributively. The input rank distribution can be either predefined or estimated via statistical inference using the received batches. The channel conditions of the outgoing links can be the packet loss rate when losses are independent. The approximation schemes of adaptive recoding proposed in References [44,79] do not require the knowledge of the channel conditions of the outgoing links. In other words, adaptive recoding can be applied without the need of feedback.

The expected rank (at the next node) of a batch in terms of the rank *r* of the batch at the current node and the number of recoded packets *t* generated by the current node for this batch is called the *expected rank function*, denoted by E(r,t). We leave a discussion on the expected rank functions to Appendix B, which also includes an example of transforming a packet loss model to suit the framework of adaptive recoding.

The model proposed in Reference [44] is a non-linear integer programming problem which groups the batches into blocks and optimizes jointly the number of recoded packets for each batch in a block. Each batch is assigned a deterministic number of recoded packets. In general, integer programming problems are NP-hard. Nevertheless, a greedy algorithm was proposed in Reference [44] to solve the problem efficiently by capturing the characteristic of the expected rank functions when the packet loss events are independent and the recoding field size tends to infinity.

Let L be the set of batches in a block. For each batch b∈L, denote the rank and the number of recoded packets to be transmitted of this batch by $r_{b}$ and $t_{b}$ respectively. Fix the total number of recoded packets to be transmitted among all the batches in the block, which is denoted by $t_{\max}\in Z^{+}$. This value can be obtained by another optimization like in Reference [43]. The model proposed in Reference [44] is the optimization problem

$$\begin{array}{cc} \max_{t_{b}\in Z\setminus Z^{-},\forall b\in L} & \sum_{b\in L}E\left(r_{b},t_{b}\right)\\ s.t. & \sum_{b\in L}t_{b}=t_{\max}. \end{array}$$

The model proposed in Reference [30] is a linear programming problem, which avoids the integer programming formulation by assigning probability distributions on the number of recoded packets to the batches conditioning on their ranks. The probability masses are variables under this relaxation. To avoid an infinite many number of variables, an artificial upper bound on the number of recoded packets of any batch is crafted. This approach is inspirited by the observation that we should not assign too many recoded packets to a batch. Although the model in Reference [30] considers only independent packet loss, the linearity of the problem preserves when we change the expected rank functions. For example, a Gilbert-Elliott model [80,81] for burst packet loss was considered in Reference [79] by modifying the linear programming problem.

Let $\left(h_{0},h_{1},\ldots ,h_{M}\right)$ be the input rank distribution, where *M* is the maximum batch size among all the batches. Further let $t_{\mathrm{avg}}\in R^{+}$ be the average number of recoded packets to be transmitted among all the batches contributed in the input rank distribution, and let $\bar{t}\in Z^{+}$ be the artificial upper bound on the number of recoded packets to be transmitted for any batch. Denote by $\alpha_{t|r}$ the probability mass of transmitting *t* recoded packets for a batch when the rank of the batch is *r*. For all *r*, we have $\alpha_{t|r}=0$ for all $t>\bar{t}$. The model in Reference [30] is the optimization problem

$$\begin{array}{cc} \max_{0\leq \alpha_{t|r}\leq 1,\forall r,t} & \sum_{r=0}^{M}h_{r}\sum_{t=0}^{\bar{t}}\alpha_{t|r}E\left(r,t\right)\\ s.t. & \sum_{r=0}^{M}h_{r}\sum_{t=0}^{\bar{t}}\alpha_{t|r}t=t_{\mathrm{avg}}\\ \; & \sum_{t=0}^{\bar{t}}\alpha_{t|r}=1,\forall r. \end{array}$$

The above two models of adaptive recoding schemes were unified in Reference [82] when the expected rank functions E(r,t) are concave with respect to *t*. The unified model is similar to the one in Reference [44] but it becomes a concave optimization problem by performing *linear interpolations* on the discrete expected rank functions.

Let $t_{r}\geq 0$ be the number of recoded packets to be transmitted when the rank of the batch is *r*. For non-integer $t_{r}$, it means that we first transmit $\left\lfloor t_{r}\right\rfloor$ recoded packets, and then we transmit one more packet with probability $t_{r}-\left\lfloor t_{r}\right\rfloor$, which is the decimal part of $t_{r}$. The unified model (It was shown in Reference [73] that this model can be extended to wireless relay networks by modifying the meanings of input rank distribution and expected rank functions) is

(2) $$\begin{array}{c} \begin{array}{cc} \max_{t_{r}\geq 0,\forall r} & \sum_{r=0}^{M}h_{r}E\left(r,t_{r}\right)\\ s.t. & \sum_{r=0}^{M}h_{r}t_{r}=t_{\mathrm{avg}}. \end{array} \end{array}$$

The formulation of this problem is similar to a *network utility maximization (NUM) problem* [83,84,85]. The traditional NUM in this form is usually for multiple users in a network, but in our case it is for a single user at a single link only. Compared to the traditional NUM, each rank corresponds to a user and the expected rank functions correspond to the utility functions of the users. This suggests that the adaptive recoding scheme for a network with multiple users is far more complicated than a NUM.

Regarding the optimal solution, Reference [82] showed that there is a robust solution which can tolerate inaccuracy on the input rank distribution. Also, there is a robust solution where the number of recoded packets is *almost deterministic*, that is, there is at most one rank such that the batches of this rank have a choice to transmit one more recoded packet. This solution is called a *preferred solution*. The optimal solution given by a solver may not be a preferred solution, so Reference [82] provided some tuning schemes which can tune any feasible (not necessary optimal) solution into a preferred solution.

Note that the objective function is not differentiable at some points. Specifically, the expected rank functions are not differentiable at the integer points. Also, the optimal solution of the optimization problem is not necessary unique. It is unclear whatever an arbitrary solver can converge to an optimal solution, although the tuning schemes can amend the output of a solver into an optimal one. We will show in Appendix C that a commonly used dual-based solver which applies a subgradient searching method is instead globally asymptotically stable, that is, it can converge to an optimal Lagrange multiplier from an arbitrary starting point.

In order to use adaptive recoding schemes, we need to calculate the rank of the received batches so that we can decide how many recoded packets are to be generated and transmitted according to the optimization problems described above. In other words, we need to perform Gaussian elimination to the batches, which is computationally expensive. If the recoding field size is large enough, the number of received packets of a batch can be an good approximation to its rank when we know the rank of this batch at the previous node.

### 3.3. Systematic Recoding

The *systematic recoding scheme* [5,41] makes use of the fact that the received packets are actually recoded packets generated by the previous node. These received packets are considered as recoded packets of the current node directly, that is, the current node simply store-and-forward these packets. The computational cost of recoding reduces significantly when the number of received packets is large. As we have mentioned in the introduction, recoding is a re-encoding procedure. This technique is called systematic recoding because its behaviour is the same as that of systematic encoding, which uses the input directly as part of the output.

In the simple protocol BATS-pro1 [41], we need to receive all the receivable packets of a batch before we can perform recoding on it. When systematic recoding scheme is applied, those received packets can be output before the reception of all the recoded packets of the batch.

A variation of systematic recoding is to drop the recoded packets of a batch which are linearly dependent of the already received packets of the same batch. Then, we can ensure that every systematic recoded packet is a useful packet. The detection of linearly dependent packets can be done together with the rank calculation of adaptive recoding if it is applied. Note that the linear dependency is preserved when we apply Gaussian elimination (by elementary column operations) on the packets, that is, the column rank of the matrix formed by juxtaposing the coefficient (column) vectors of the packets is the same as the column rank after transforming the matrix into the column echelon form. So, we can modify the packets in place as an implementation of systematic recoding together with the detection of linear dependency.

The analysis of systematic recoding schemes can be found in Reference [5]. In the analysis, the theoretical throughput is better for systematic recoding schemes than the strategy that generates all the recoded packets by RLNC. However, this difference in the throughput is too small to be observed in the simulation results.

**Figure 1:**
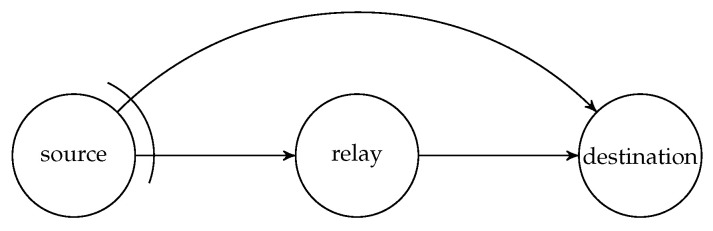
A wireless relay network.

Although there are benefits of using systematic recoding schemes, it is not suitable for all network models, for example, the wireless relay network illustrated in Figure 1 where the source node broadcasts the packets to the other two nodes. Here, the link between the source and destination nodes is an overhearing channel. The deployment of BATS codes in wireless relay networks was discussed in Reference [73]. There is a chance that both the relay and destination nodes receive the same packet transmitted by the source node. If the relay node adopts a systematic recoding scheme, then a packet already received by the destination node directly from the source node may be transmitted again by the relay node, which is obviously redundant. This can be prevented by using RLNC to generate all the recoded packets.

### 3.4. Causal Recoding

*Non-causal recoding* refers to generating recoded packets after all the receivable packets for a particular batch are received. Instead of idling when a node is waiting for the reception of additional packets to perform non-causal recoding, we can fully utilize the channel by transmitting recoded packets generated from the already received packets. That is, we allow the generation of recoded packets of a batch before we confirm that all the receivable packets of the batch are received (This idea was first presented in the tutorial “BATS Codes: Theory and Practice” at 2018 IEEE International Symposium on Information Theory, Vail, Colorado, USA). A more general strategy is that we do not allow the generation of recoded packets before a certain *threshold* on the rank or the number of received packets in a batch is reached. We call this approach *causal recoding*.

It is easy to see that we can enhance the throughput by transmitting extra causally recoded packets beyond those non-causally recoded packets. On the other hand, compared with transmitting non-causally recoded packets only, if we transmit the same number of recoded packets but with some of them being causally recoded, the throughput can potentially drop. To see this, we only need to demonstrate that the information carried by the batches is more likely to be lost.

Suppose we use RLNC to generate the recoded packets. Let C be the matrix formed by juxtaposing the received packets. Let T, called the *recoding matrix*, be the matrix containing the coefficients of the linear combinations performed by the recoding procedure. Then, the juxtaposed recoded packets is the matrix CT.

An example of the matrix T for a causal recoding scheme with a threshold of one received packet is

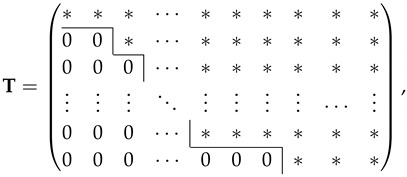

where the asterisks are i.i.d. elements over the recoding field as the recoded packets are generated by RLNC. Here, each column of T corresponds to a recoded packet, where the asterisks in the column indicate those received packets that contribute to the recoded packet. Likewise, each row of T corresponds to a received packet, where the asterisks in the row indicate which recoded packets the received packet contributes to. We can see that when we go down the rows from the top, the received packet contributes to fewer recoded packets, so that the information about that packet is more likely to be lost. For example, suppose the rightmost received packet in C is linearly independent of all other received packets in C. For the matrix T shown above, we would lose all the information about the rightmost packet in C if the three rightmost recoded packets in CT are lost.

For RLNC that applies non-causal recoding, the recoding matrix T is a totally random matrix over the recoding field, so that every entry of T is an asterisk. As such, the information about a received packet is completely lost only when all the recoded packets are lost.

Despite such a drawback, we still consider causal recoding schemes because the threshold is actually a trade-off between throughput and delay. The latter is a concern for real-time applications when applying BATS codes.

## 4. BATS Protocol Modules

In order to distinguish the packets in the theory of BATS codes from those with suitable headers for network transmission, we coin the following terminologies.

**Definition** **8.** *A packet freshly generated by the BATS encoder without the coefficient vectors and other headers is called a raw packet.*

**Definition** **9.** *A packet which includes the coefficient vectors and other headers for a BATS protocol with the raw packet as the payload is called a BATS packet.*

Yet, when there is no confusion or it is not necessary to distinguish the two types of packets, we would simply call them the packets. When the raw packets in a batch are converted into BATS packets, we continue to call this set of BATS packets a batch for convenience.

There are three main modules in a BATS protocol: the *encoding module*, the *batch forwarding module* and the *decoding module*. The encoding module applies a BATS encoder on the data to be encoded, converts the raw packets into BATS packets, and outputs the BATS packets. The batch forwarding module performs recoding on the batches and outputs the BATS packets of different batches in a specific order, for example, in a sequential or interleaved order. The decoding module gathers the BATS packets, converts them back into raw packets, and applies BATS decoding to recover the data.

In this section, we present the idea and design of the three modules of a BATS protocol which can use arbitrary recoding schemes and burst loss handling techniques. We leave the discussion on the design of BATS packets to the next section.

### 4.1. Batch Stream and Interleaver

**Figure 2:**
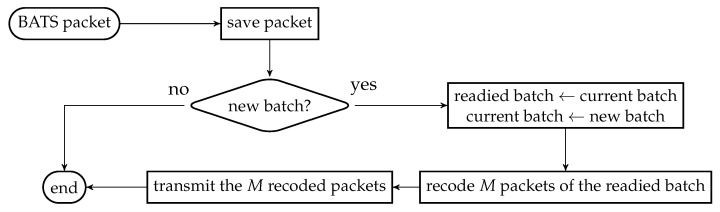
Flowchart of the batch forwarding module of the protocol BATS-pro1 at an intermediate node.

We first reproduce in Figure 2 the flowchart for the batch forwarding module of BATS-pro1 [41] to facilitate our discussion of an interleaved version of BATS-pro1. BATS-pro1 applies the baseline recoding scheme, so we generate the same number of packets for all batches. In the flowchart, *M* is the number of recoded packets to be generated.

**Figure 3:**
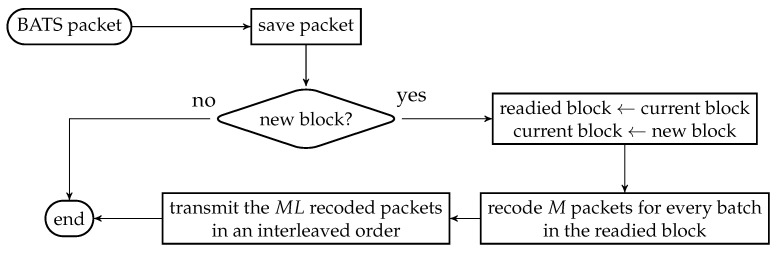
Flowchart of the batch forwarding module of interleaved BATS-pro1 at an intermediate node.

As the design generates the same number of packets for all batches, a block interleaver is a suitable choice to interleave the batches. Let *L* be the depth of the block interleaver. We group *L* batches into a *block*. The flowchart of the batch forwarding module of interleaved BATS-pro1 shown in Figure 3, which is reproduced from Reference [86], is a minor modification of the one for BATS-pro1.

**Figure 4:**
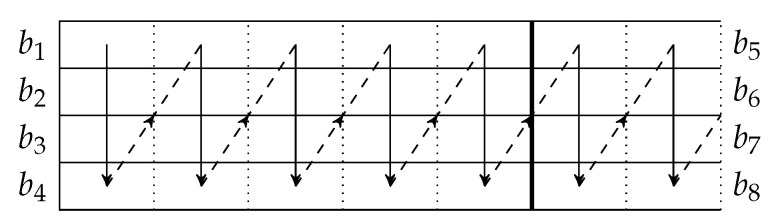
The transmission sequence of interleaved BATS-pro1 where M=5 and L=4. Each rectangle represents a batch and its length represents the number of recoded packets.

By concatenating the blocks to be transmitted, we can use a transmission sequence like the example shown in Figure 4. In the figure, $b_{1}$, $b_{2}$, *…* represent the batches, where $b_{1}$ to $b_{4}$ belong to a block, $b_{5}$ to $b_{8}$ belong to another block, and so forth. We can see that batch $b_{i+4}$ can be regarded as logically appended to $b_{i}$ for i=1,2,… If we assume that there is no out-of-order packets, then once we receive a packet from batch $b_{i+4}$, we can be assured that all the receivable packets of batch $b_{i}$ have been received.

**Figure 5:**
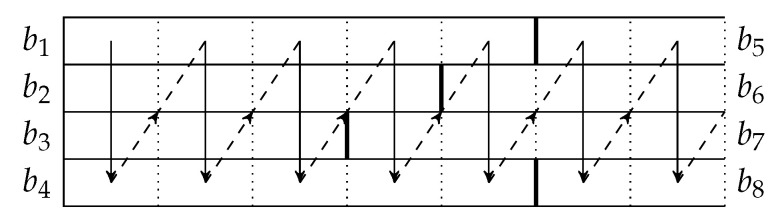
An example of the transmission sequence of 4 batch streams where each batch can have different numbers of recoded packets. Each rectangle represents a batch and its length represents the number of recoded packets.

In Figure 4, $b_{1}$, $b_{5}$, $b_{9}$, *…* form a *batch stream*, and so do $b_{2}$, $b_{6}$, $b_{10}$, *…* Thus for an interleaver of depth *L*, there are *L* batch streams. In general, there can be different numbers of recoded packets in the batches. An example of the transmission sequence for such batches is illustrated in Figure 5.

We have seen how the batch streams are formed for transmission. At an intermediate node, the batch streams need to be reconstructed so that the node can detect the last receivable packet of a batch timely and decide when to discard that batch.

### 4.2. Encoding Module

**Figure 6:**
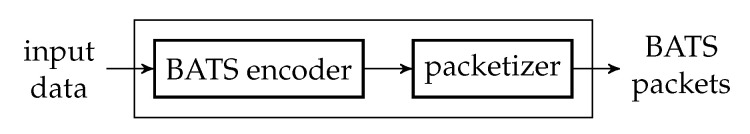
An encoding module.

An encoding module is a wrapper of a BATS encoder where the BATS encoder is accompanied by a packetizer. There can be more than one encoding module at a network node. Figure 6 illustrates an encoding module.

The number of raw packets in a batch output by the BATS encoder is the batch size of this batch, and these raw packets are regarded as linearly independent of each other by the inner code. The batches are then passed to the packetizer, which prepends the headers so that the batches now contain BATS packets which are ready to be transmitted. The packetizer then outputs a single batch stream which is formed by concatenating the batches sequentially.

After enough batches for the input data of an information source are generated, we replace the BATS encoder by a new one for the encoding module so that it can encode the input data of another information source. If there is not another information source after a timeout, we should transmit dummy packets to indicate the end of transmission. The reason for using dummy packets will be discussed in Section 4.4.1.

### 4.3. Decoding Module

**Figure 7:**
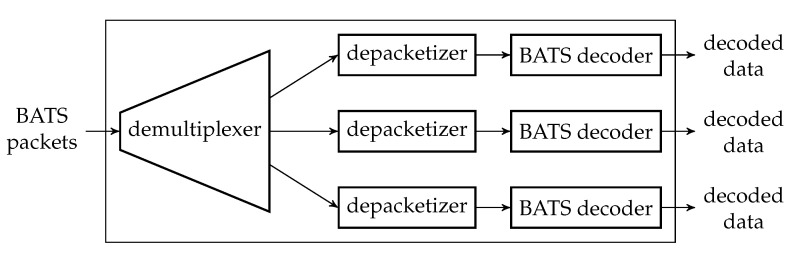
An example of a decoding module having three BATS decoders.

The decoding module is a wrapper of at least one BATS decoder and there can be more than one such module at a network node. Figure 7 illustrates an example of a decoding module.

Before a BATS packet arrives at a BATS decoder, it has to pass through a depacketizer which converts BATS packets into raw packets. The raw packets of the input data of an information source must arrive at the BATS decoder for this information source. Therefore, we need a demultiplexer to distribute the BATS packets to the correct depacketizer which connects to the correct BATS decoder. The demultiplexers can also be implemented outside the decoding modules.

Note that a BATS decoder can accept the raw packets in an arbitrary order, so it is not necessary to deinterleave the packets even if they are interleaved.

### 4.4. Batch Forwarding Module

The batch forwarding module is the most complicated one among the three basic modules. There are four types of submodules inside a batch forwarding module, which are the *input stream management unit (ISMU)*, the *recoding unit*, the *batch buffer* and the *output stream management unit (OSMU)*.

Each ISMU is responsible for an incoming link. Similarly, each OSMU is responsible for an outgoing link. Note that a link can be a virtual link within a node. For example, we can have a virtual link between an encoding module and a batch forwarding module so that the BATS packets output by the encoding module are regarded as the input to an ISMU in the batch forwarding module.

The incoming BATS packets are passed to the ISMU, which handles out-of-order packets and indicates the reception state of the batches. The packets are then stored in the batch buffer. The batch buffer is not just a storage for batches, but it also requests recoded packets from the recoding unit. On the other hand, the OSMU requests batches from the batch buffer, manages batch streams for transmission and outputs BATS packets in a specific order. We can also include a rate control mechanism in the OSMU.

**Figure 8:**
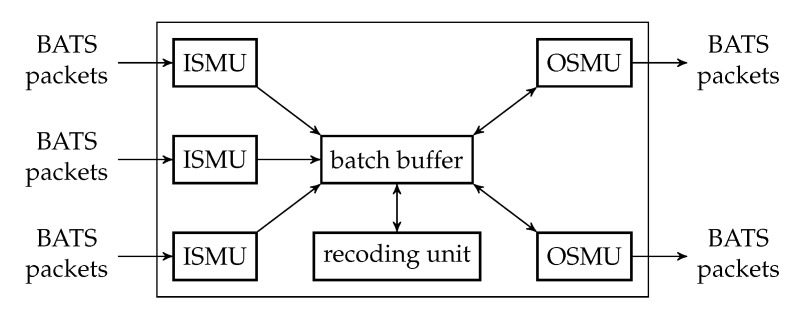
An example of the relation between the submodules inside a batch forwarding module.

An example of the relation among the submodules of a batch forwarding module is illustrated in Figure 8 where there are three incoming links and two outgoing links.

#### 4.4.1. Input Stream Management Unit (ISMU)

The ISMU has the following functions:

1. recognize different batch streams from the incoming packets;
2. identify batches for which all receivable packets have been received;
3. handle broken batch streams; and
4. handle out-of-order packets.

The sequence of the input BATS packets depends on the transmission sequence of the previous node and the packet loss pattern of the incoming link. Note that even if the previous node transmits BATS packets in a constant rate, the current node cannot detect the lost packets by timing the intervals between any two consecutively received BATS packets when there are packet jitters.

**Figure 9:**
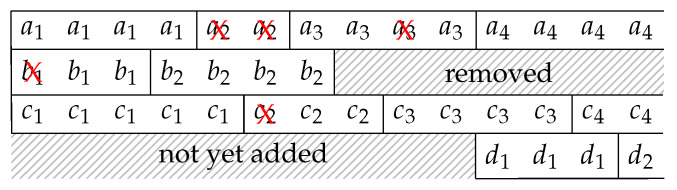
An example of the batch streams at the output stream management unit (OSMU) at the previous node.

We give an example to illustrate the reconstruction of the batch streams. Suppose the batch streams at the OSMU at the previous node are shown in Figure 9. At the beginning, there are only three batch streams. The OSMU may change the interleaver depth at any time by removing or adding batch streams. In the figure, we use *a*, *b*, *c* and *d* to represent the four batch streams and use integer subscripts to identify the batches of the streams, for example, $a_{1}$ is the first batch in batch stream *a*. In this example, the second batch stream is removed after transmitting two batches, and a new batch stream shown in the last row of the figure is added during the transmission. We denote the BATS packets of a batch by the identification of the batch, for example, there are four BATS packets in batch $a_{1}$ so we have four $a_{1}$’s in the figure. A cross on a BATS packet means that that packet is not received by the current node.

Suppose the OSMU of the previous node outputs the BATS packets in a round-robin manner among the batch streams. Although it is not required to receive the BATS packets right after the previous node starts the transmission, we assume that it is the case in this example for demonstrating the idea of the ISMU. Then, the packet reception sequence at the current node is

$$a_{1}c_{1}|a_{1}b_{1}c_{1}|a_{1}b_{1}c_{1}|a_{1}b_{2}c_{1}|b_{2}c_{1}|b_{2}|a_{3}b_{2}c_{2}|a_{3}c_{2}|c_{3}|a_{3}c_{3}|a_{4}c_{3}d_{1}|a_{4}c_{3}d_{1}|a_{4}c_{4}d_{1}|a_{4}c_{4}d_{2}|\ldots ,$$

where we use vertical bars to separate the sequence so that it is easier to read. Those vertical bars are not part of the sequence and thus they are not identified by the current node.

In BATS-pro1, since there is only one batch stream, upon receiving a packet of a new batch, we know that all the receivable packets of the previous batch have been received. When there are multiple batch streams, the same can be achieved for a particular batch stream by including in the BATS packets the identification of the previous batch. This will be discussed in Section 5.

**Figure 10:**
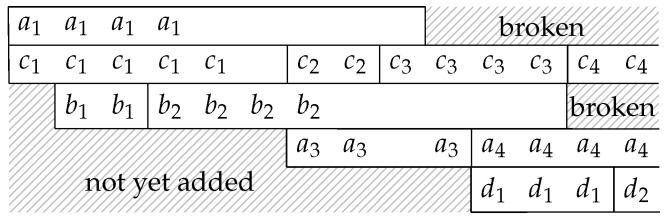
An example of batch streams reconstruction at the input stream management unit (ISMU).

In the example of the batch stream reconstruction in Figure 10, we assume that the identification of the previous batch is known. We initialize the ISMU with no batch stream in it. At any subsequent time, when we receive a BATS packet where its previous batch is not in any reconstructed batch streams, we start a new batch stream with that batch. In Figure 9, all the BATS packets of batch $a_{2}$ are lost. Upon receiving the packets of batch $a_{3}$, we start a new batch stream with this batch. The ISMU does not know that batch $a_{1}$ is in the same batch stream, and there is no way the ISMU can tell that all the receivable packets of batch $a_{1}$ have already been received. To handle this kind of issues, we need to define a timeout so that a batch stream is considered *broken* when no BATS packet in the batch stream is received after the timeout. A broken batch stream can be removed from the reconstructed batch streams. By applying the rules mentioned above, the reconstructed batch streams are shown in Figure 10.

The batch stream after batch $b_{2}$ is meant to be broken as it is decided by the previous node. A minor improvement is that *dummy packets* which act as a batch following batch $b_{2}$ can be transmitted by the previous node, so that the batch stream can be identified as broken earlier. The design of dummy packets will be discussed in Section 5.2.4.

Here is a brief summary on the above discussion.

1. The BATS packets of a batch have to include the identification of the previous batch in the same batch stream, which should be done by the OSMU at the previous node.
2. For a BATS packet where its previous batch is not in the reconstructed batch streams, the batch the packet belongs to is added to a new batch stream.
3. A timeout is set so that a batch stream without any further BATS packets received within a certain period is marked as broken, and such a broken batch stream will be removed shortly.
4. Dummy packets can be transmitted before the removal of a batch stream at the previous node so that the current node can mark a batch stream as broken before the timeout.

If the current batch is the first batch in a batch stream, then there is no previous batch for this batch. The design for the identification of the previous batch of such a case will be discussed in Section 5.2.4.

**Figure 11:**
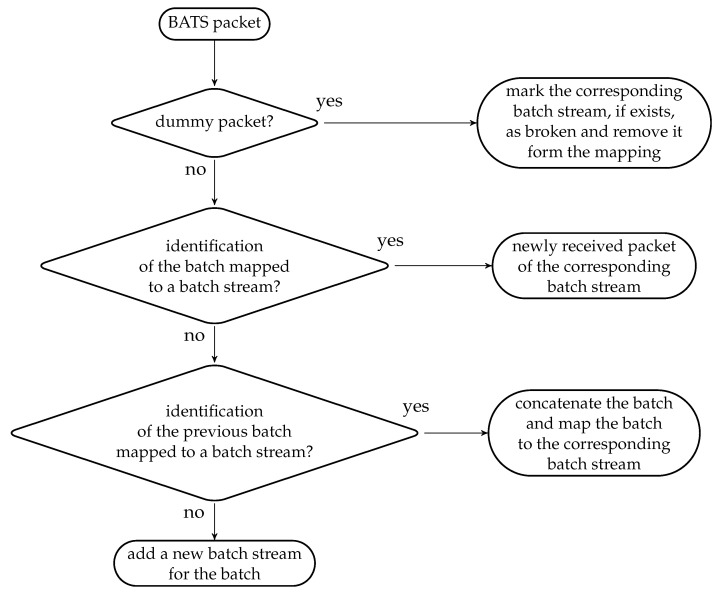
Flowchart for the batch stream detection process by the ISMU.

The received BATS packets are stored in the batch buffer so the ISMU only needs to keep track of the identifications of the latest batches in the batch streams. The ISMU maintains a *bijective mapping* between the identifications of the batches and the batch streams. The batch stream detection process has the following steps, which is also illustrated as a flowchart in Figure 11:

1. if the BATS packet is a dummy packet, then the corresponding batch stream is marked as broken and removed from the mapping; else
2. if the identification of the batch is mapped to a batch stream, then the BATS packet is a newly received packet of the corresponding batch stream; else
3. if the identification of the previous batch is mapped to a batch stream, then the batch the BATS packet belongs to is concatenated to that batch stream and the identification of the batch is mapped to that batch stream; otherwise
4. a new batch stream is added for the batch.

As an example, if we number the batch streams shown in Figure 10 from top to bottom starting from 1, then after the ISMU has handled the latest received packet, that is, a BATS packet from batch $d_{2}$, the bijective mapping is the one shown in Table 3 below. Note that we can reuse the first batch stream for batches $d_{1}$ and $d_{2}$ so that we do not need to add a new batch stream for them, but this depends on the implementation.

When a batch is concatenated to a batch stream, the previous batch in the same batch stream is identified as one for which all the receivable packets have been received. Similar for the latest batch in a broken batch stream. However, this identification is erroneous if there are out-of-order packets across the batches, which may be due to wireless signal reflection or the BATS packets of a batch arriving at the node from different incoming links. Note that the batch stream detection process puts the first out-of-order packet of a batch in a new batch stream. Although the BATS packets of a batch may be distributed to more than one batch stream, these packets are eventually merged into a single batch in the batch buffer.

#### 4.4.2. Batch Buffer

The basic function of the batch buffer is to provide a temporary storage for the received BATS packets and recoded packets of the batches. In addition, the batch buffer provides the following functions:

1. groups the packets according to the identifications of the batches they belong to;
2. requests recoded packets from the recoding unit and outputs them when requested by the OSMU(s); and
3. records statistical data for each batch, such as the rank, the number of received packets, the number of BATS packets queried by the OSMU(s), the arrival timestamp, and so forth.

The batch buffer only outputs recoded packets of a batch to an OSMU such that the destination of this batch can be reached through the link this OSMU is responsible for.

We design three flags for each batch, namely

1. the *finished* flag: this flag is marked by the ISMU after it has confirmed that all the receivable packets of this batch are received, so the recoded packets generated for this batch after the finished flag is marked are non-causally recoded packets;
2. the *active* flag: this flag is marked by the OSMU, which means that some BATS packets of this batch have already been assigned to some output batch streams, that is, those BATS packets have already been transmitted or pending to be transmitted; and
3. the *recoded* flag: this flag is marked by the recoding unit after a sufficient number of non-causally recoded packets have been generated for this batch, so that this batch can be removed from the batch buffer after a timeout which starts after all the recoded packets of this batch have been transmitted.

Every new batch is marked as *unfinished*, *inactive* and *non-recoded*. An *arrival timestamp* is recorded when the first BATS packet of a new batch is added to the batch buffer.

The batch buffer requests the remaining recoded packets of a batch from the recoding unit after the finished flag is marked for this batch, which avoids the need of real-time computation when a recoded packet is requested. Due to the linearity of RLNC, we can combine a newly received packet to the existing recoded packets without recalculating all the linear combinations. If it happens often that we receive additional BATS packets of a batch after it is marked as finished, then we can consider delaying the marking of the finished flag by a timeout. That is, if the ISMU wants to mark a finished flag of a batch and there is no more BATS packets received for this batch after the timeout, then the batch buffer marks the flag for the batch.

The timeout for the batch removal mentioned in the description of the recoded flag is for the BATS packets of this batch which are received after all the other recoded packets of this batch are transmitted. The handling of these BATS packets depends on the transmission policy. For example, we can regard these packets as being in a new batch, but we include the already transmitted packets when we generate the recoded packets by RLNC.

When the OSMU requests a BATS packet of a batch given the identification of the batch, the batch buffer

1. outputs a recoded packet in its storage if there is any; otherwise
2. requests a recoding packet from the recoding unit and outputs it.

**Figure 12:**
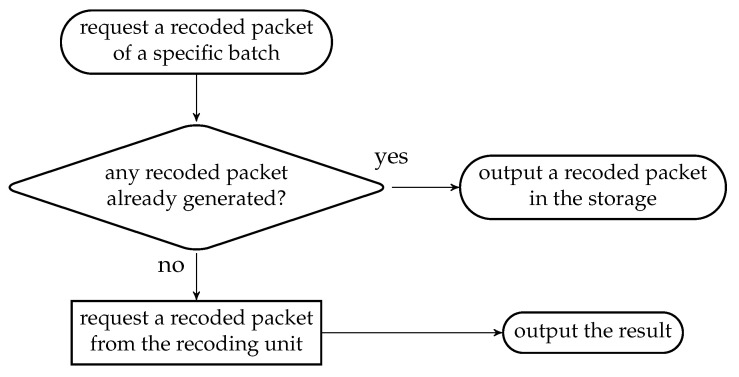
Flowchart for the selection of a recoded packet by the batch buffer when requested.

The flowchart of the above steps is illustrated in Figure 12. If the recoding unit outputs nothing, then the batch buffer also outputs nothing.

We can see that the BATS packets are added to the batch buffer one by one, but the batch buffer removes the BATS packets batch-by-batch. One possible implementation of the batch buffer is to use linked lists as follows.

We first decide the maximum number of BATS packets the batch buffer can store, allocate the necessary amount of memory and arrange the memory into a linked list. Initially, this linked list consists of the *unused slots* in the batch buffer. When a BATS packet is put into the batch buffer, the first unused slot is removed from the linked list and this linked link node is appended to the corresponding linked list for the batch this packet belongs to.

**Figure 13:**
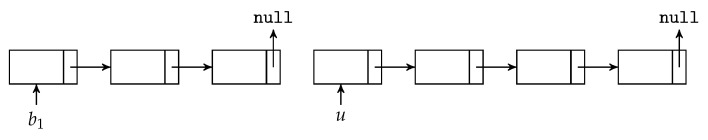
An example of the linked lists for the unused slots and a batch $b_{1}$.

**Figure 14:**
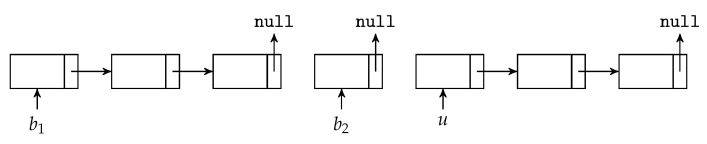
An example of the linked lists following Figure 13 after a BATS packet of a new batch $b_{2}$ is put into the batch buffer.

As a graphical illustration, suppose the linked lists before we add a BATS packet into the batch buffer are shown in Figure 13, where *u* and $b_{1}$ in the figure represent the entry points of the linked lists for the unused slots and a batch $b_{1}$ respectively. When we put a BATS packet of a new batch $b_{2}$ into the batch buffer, it becomes three linked links as shown in Figure 14.

**Figure 15:**
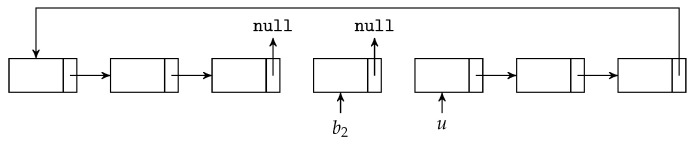
An example of the linked lists following Figure 14 after the removal of batch $b_{1}$ from the batch buffer.

When we remove a batch from the batch buffer, we can append the linked link of this batch to the linked list for unused slots. For example, Figure 15 illustrates the removal of the batch $b_{1}$ from the linked lists shown in Figure 14. In this way, both adding a BATS packet and removing a batch can be done in constant time.

#### 4.4.3. Recoding Unit

Similar to the batch buffer, we can have more than one recoding unit at a node. A recoding unit handles tasks related to recoding, which includes

1. the generation of recoded packets; and
2. the calculation of the number of recoded packets to be generated for the batches.

**Figure 16:**
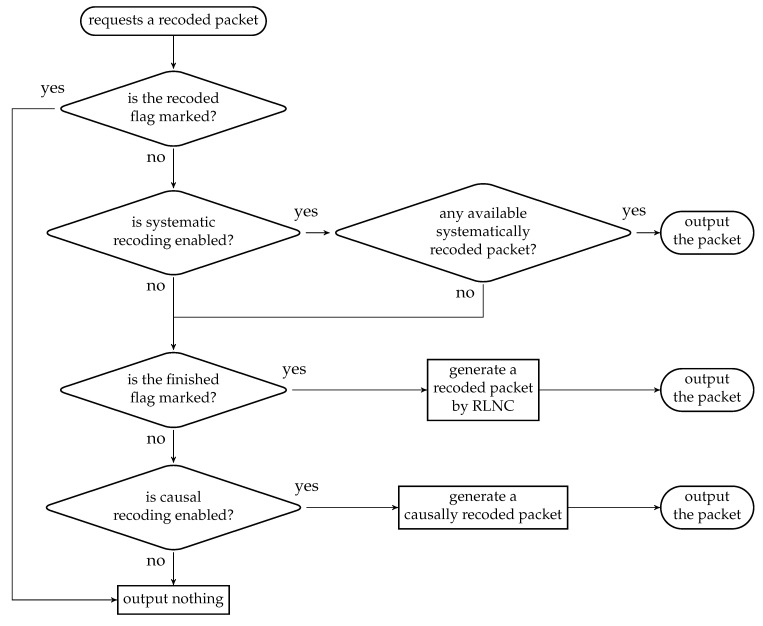
Flowchart of an example on deciding which recoding scheme to use to generate a recoded packet.

For the first task, the way to generate a recoded packet depends on the recoding scheme unless a causally recoded packet is requested specifically. One possible way for making the decision is illustrated in Figure 16, which consists of the following steps:

1. if the recoded flag is marked, then output nothing; else
2. if systematic recoding is enabled and there are received BATS packets in the batch buffer not previously output as a systematically recoded packet, then output one such packet; else
3. if the finished flag is marked, then generate a recoded packet by RLNC; else
4. if causal recoding is enabled, then generate a causally recoded packets by RLNC; else
5. output nothing.

If baseline recoding is used, then the second task is trivial as the number of recoded packets is predefined. Otherwise, when adaptive recoding is used, we need to solve the corresponding optimization problem described in Section 3.2. The input rank distribution for the optimization problem can be either given beforehand or estimated during the reception of batches. The ranks of the batches are recorded in the batch buffer so that we can obtain the number of recoded packets to be generated. Once we have generated enough recoded packets for a batch, the recoded flag of the batch is marked in the batch buffer.

There are scenarios that the recoding unit needs to modify the recoded packets which are generated already. For example, the batch buffer invokes the recoding unit to generate recoded packets after the finished flag is marked but then an out-of-order packet is received. In this case, each of the recoded packets which is not systematic and not yet transmitted is read from the batch buffer and modified by adding to it the newly received packet multiplied by a randomly chosen scalar in the recoding field.

#### 4.4.4. Output Stream Management Unit (OSMU)

There can have multiple OSMUs deployed at a network node, for example, one OSMU for one network interface card (NIC). The OSMU has the following functions:

1. assign batches in the batch buffer to construct batch streams;
2. transmit the BATS packets in the batch streams in a specific order; and
3. request recoded packets from the batch buffer.

Unlike the ISMU which only needs to keep track of the identifications of the batches, the OSMU needs to form instances of batch streams. A batch stream in the OSMU is a queue of BATS packets which are ready to be transmitted. The number of batch streams is related to the transmission sequence, for example, the number is the interleaver depth if we transmit the batch streams in a round-robin manner. This number can be changed over time in order to adapt the channel conditions of the outgoing links when necessary. The criteria for changing the number of batch streams and the transmission rate are not discussed in this paper as they are independent of the design paradigm.

When the OSMU is going to transmit a BATS packet in a batch stream but there is no BATS packet in that batch stream, the OSMU requests from the batch buffer a recoded packet of the latest batch in that batch stream. If the batch buffer outputs nothing, then we have two cases:

1. if the corresponding batch is marked as recoded, then it means that the OSMU has to select another batch for this batch stream; otherwise
2. the batch buffer is waiting for more BATS packets of this batch.

For the first case, the OSMU triggers a procedure which selects another batch and concatenates it to the batch stream. We will discuss this procedure later. For the second case, the OSMU can take different actions according to the transmission policy, for example,

- trigger the aforementioned procedure to select a new batch and transmit its BATS packets, and transmit the out-of-order BATS packets of the original batch later;
- transmit a BATS packet of a batch in another stream;
- give up the chance to transmit a BATS packet.

**Figure 17:**
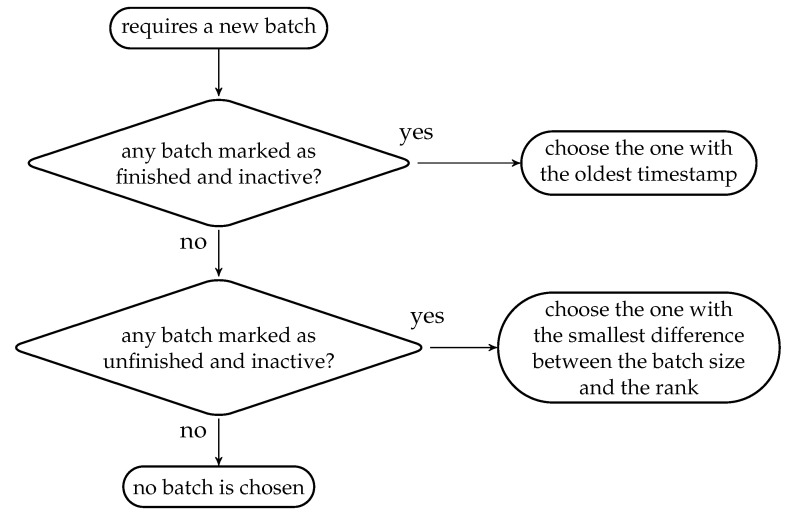
Flowchart of the procedure when a new batch is required to be concatenated to a batch stream in the OSMU.

We now describe the procedure for selecting a batch to be concatenated to a batch stream, which is illustrated in Figure 17. When a batch is selected from the batch buffer, batches marked as finished and inactive have higher priority than batches marked as unfinished and inactive. We set this priority because the batches marked as finished have potentially received all receivable packets so that they are ready to be recoded and transmitted. We only consider batches marked as inactive as these batches are not part of another batch streams.

Among those batches marked as finished and inactive, we select the one with the oldest timestamp to prevent inducing more delay to it. For those batches marked as unfinished and inactive, we choose the one with the smallest difference between the batch size and the rank, because intuitively this batch has a higher chance to be marked as finished soon. We consider the difference but not the batch with the highest rank here because the latter strategy has a bias that tends not to select the batches with smaller batch sizes. However, both strategies are equivalent if all batches have the same batch size. If we know the ranks of the batches at the previous node, then we can instead compare the difference between the rank of a batch at the previous node and the rank of the same batch at the current node.

If there is no inactive batch in the batch buffer, then we can either transmit nothing or transmit a causally recoded packet of a batch in another batch stream if there is any. The former action can make the outgoing link available for the use by other programs in the same system so that the OSMU will not make the overall environment unfair, while the latter action can potentially reduce the packet loss of some other batches. We choose to transmit a causally recoded packet here because a normally recoded packet would implies a shorter time between two consecutive transmission of the batch which means that the batch becomes more vulnerable to burst packet loss.

### 4.5. Trivial Example on Assembling the Modules

**Figure 18:**
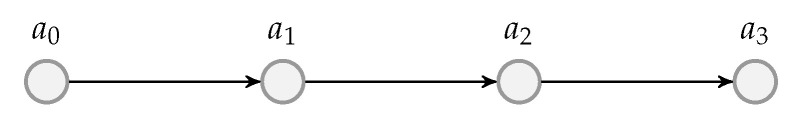
A three-hop line network.

Line network is a fundamental building block of a network so it is used to demonstrate BATS codes in various works including References [30,44,46,79]. In a line network, network links only exist between two neighboring nodes. As an example, Figure 18 illustrates a three-hop line network, where the nodes $a_{0}$ and $a_{3}$ are the source and destination nodes respectively.

**Figure 19:**
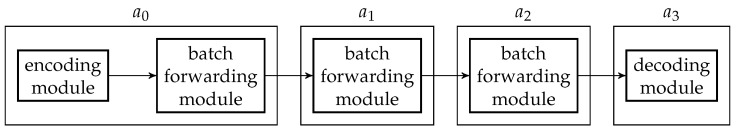
The assembly of the modules of BATS protocol in a three-hop line network having single directional traffic flow only.

We demonstrate in Figure 19 the simplest case to assemble the modules in the line network shown in Figure 18. We will discuss more about the assembly of the modules in Section 6. A trivial way is to apply an encoding module at the source node, a decoding module at the destination node, and batch forwarding modules at all the nodes except the destination node. We connect the encoding module to a batch forwarding module at the source node so that recoding and interleaving can be applied to the batches at the source node. We can generate more BATS packets for the batches at the source node so that this redundancy can reduce the drop of the ranks of the batches. It was demonstrated in References [30,73] that a suitable redundancy can improve the throughput and packet efficiency.

**Figure 20:**
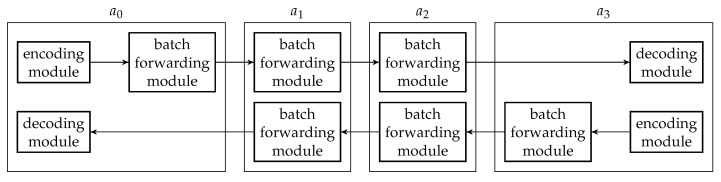
The assembly of the modules of BATS protocol in a three-hop line network having bidirectional traffic flow.

If the traffic is bidirectional, that is, there is another flow of traffic from node $a_{3}$ to $a_{0}$, then we only need to duplicate the modules in a similar way as shown in Figure 20. Depending on the configuration, we can merge the two batch forwarding modules within the same node into one.

## 5. BATS Packet Design

In this section, we discuss the design of BATS packets and how the BATS protocol cooperates with the existing network infrastructures.

### 5.1. Packet Flow

The example we demonstrated in Figure 19 is only a single user model. An intermediate network node may generate and receive its own traffic, for example, a node in an ad hoc network. Therefore, a general BATS code enabled network node should have all the three modules of the BATS protocol in it.

**Figure 21:**
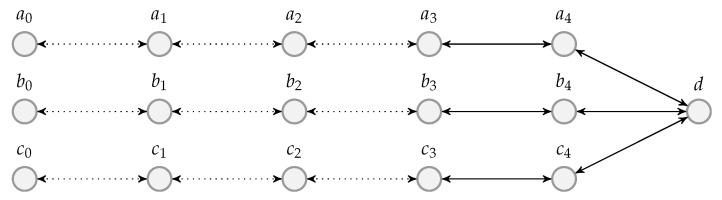
A network topology having multiple wireless and wired links. The wireless links are indicated by the dotted lines.

There is a longer delay than the traditional forwarding strategy when we apply BATS codes which is due to the recoding procedure. However, it is not necessary to apply recoding for all outgoing links. Take the network topology illustrated in Figure 21 as an example. In the figure, wireless and wired links are indicated by dotted and solid lines respectively. Suppose the wired links are stable and reliable. Nodes $a_{0}$, $b_{0}$ and $c_{0}$ are the source nodes and node *d* is the destination node. We apply BATS codes at the source nodes. We should apply recoding at the intermediate nodes $a_{i}$, $b_{i}$ and $c_{i}$ for i=1,2 if it is allowed, as it is likely for packet loss to occur at the wireless links. As we assume that the wired links are reliable, the packets are not likely to be lost even when BATS codes are not applied. If we also apply recoding at the remaining intermediate nodes $a_{i}$, $b_{i}$ and $c_{i}$ for i=3,4, it would induce extra delay but have no extra advantage.

We can also apply a BATS decoder before the data reach the destination node. If we apply BATS decoders at the destination node *d* shown in Figure 21, then we have to deploy three BATS decoders at node *d*. This design is not scalable as this centralized node for decoding can be overwhelmed. One solution is to apply BATS decoders at nodes $a_{3}$, $b_{3}$ and $c_{3}$, so that the computation for the three decoding procedures is distributed to the three nodes. This means that a piece of data can be encoded and decoded multiple times during the transmission. Note that we may need better devices at these nodes to ensure that there are enough memory and computational power to perform decoding efficiently.

**Figure 22:**
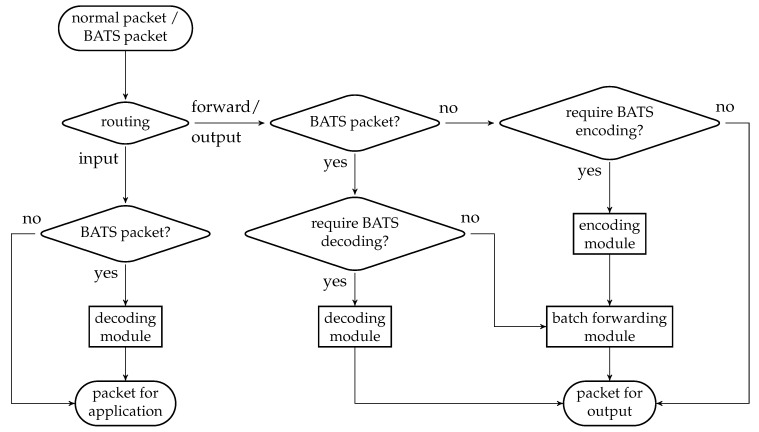
A general packet flowchart.

Following the discussion above, we present a general packet flowchart in Figure 22. We consider both packets from normal traffic and the BATS protocol. The flowchart shows a guideline where a node

1. supports the role(s) of source, destination and/or intermediate node;
2. works in conjunction with normal traffic; and
3. has a choice of applying forwarding instead of recoding.

When a BATS packet arrives at a node which is not the destination of this packet, that is, we follow the “forward” branch in the flowchart, the node has to decide whether it should pass the packet to the decoding module. Similarly, if we follow the “forward” branch in the flowchart but the packet is not a BATS packet, the node has to decide whether it should pass the packet to the encoding module. These decisions are NIC and/or subnet specific. For example, if we only apply BATS codes for the wireless links in the network shown in Figure 21, then node $a_{3}$ would decode the traffic towards node $a_{4}$ and encode the traffic towards node $a_{2}$. There may have exceptions on the decision as the change of the traffic by BATS codes may affect the multicast and broadcast loop detection mechanism. An example of this kind of traffic is the multicast packets for the multicast domain name system (mDNS) protocol.

Another decision which is imbedded in the batch forwarding module is whether the node needs to perform recoding. For example, if the outgoing link is stable and reliable, then the recoding operations can be omitted so that the overall delay is reduced. Another scenario is that the node does not have enough computational power for recoding so that it should skip the recoding operations.

In the following subsections, we further describe the branches in the packet flowchart when the BATS protocol is implemented in the transport layer and the application layer respectively.

#### 5.1.1. Transport Layer

The BATS protocol in a transport layer implementation is a payload of a network layer protocol like the Internet Protocol (IP). We assume that we use IP in the following discussion (Although we may use an unused type of the Internet Control Message Protocol (ICMP) to carry the BATS protocol, it may have an issue as mentioned in RFC 1122 ([87], Section 3.2.2) that an ICMP message of unknown type must be silently dropped). A protocol number has to be defined for the IP header so that we can distinguish the BATS protocol from other protocols. We do not need the header of another transport layer protocol like the User Datagram Protocol (UDP) hence the packet size is smaller than implementing the BATS protocol in the application layer.

Other than implementing a kernel module to handle this transport layer BATS protocol, we can use firewall rules to distinguish between normal and BATS packets and also to filter out the traffic which does not require the application of BATS codes. An issue of not using a kernel module is that we have to bring the packets to the user space, which incurs overhead on memory copying. Also, we may need the administrator right or to be in a special user group in order to be able to configure the packet filtering rules, capture the BATS packets for recoding and transmit tailor-made IP packets.

We do not need to specify the IP address of the next node if the other nodes apply filters to capture the BATS packets. A merit of this implementation is that it is independent of the internal routing table, so it is compatible to routing protocols and the network topology can be dynamic. Also, it is transparent to other applications whose traffic we want to carry via BATS codes, as their traffic can be captured and becomes the payload of the BATS protocol.

For those commonly used transport layer protocols like UDP and TCP, the behaviour when their packets pass through a network address translator (NAT) device is well-defined and supported by the native networking devices. However, for the BATS protocol, we need to define the mapping when its packets pass through an NAT device if the traffic is bidirectional. This can be an issue when the NAT device is not under our control.

**Figure 23:**
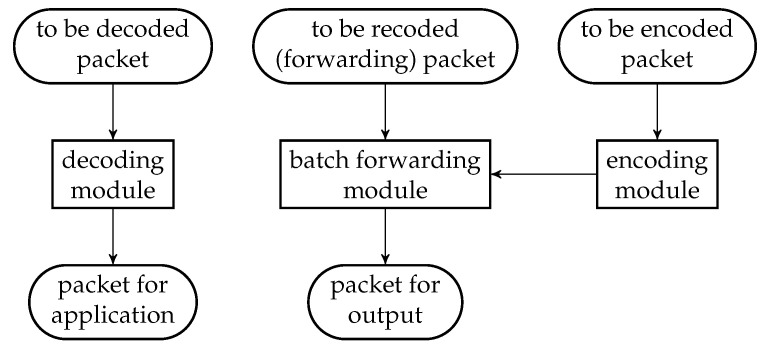
A packet flowchart for a transport layer implementation, or an application layer implementation which depends on packet filtering.

The packet flow illustrated in Figure 23 is divided and filtered into different simple flows. The traffic which bypass BATS codes are handled by the default procedure of the operating system so we do not show those cases in the flowchart.

#### 5.1.2. Application Layer

In an application layer implementation, the BATS protocol itself is a payload of a transport layer protocol like UDP. Assume that we use UDP in the following discussion. We can define a port number as an identification of the BATS protocol, which is a common practice for other application layer protocols like Network Time Protocol (NTP). Note that UDP is natively supported by the NAT devices so that we do not need extra configuration on it.

There are two different approaches for the implementation. The first approach is the same as the transport layer implementation except that the BATS protocol is now over a transport layer protocol. Like the transport layer implementation, this approach has the advantage that we do not need to specify the IP address of the next node so that it is independent of the routing mechanism and the network topology can be dynamic. Similarly, we need to have extra permission so that we can filter and capture the BATS packets for recoding. The packet flowchart for this approach is the same as the one shown in Figure 23.

The second approach is that we do not depend on packet filtering. That is, the destination IP address of a BATS packet is the next node which applies recoding or decoding. Although it is easy to implement and it works in conjunction with normal traffic by its nature, the network topology is static, or it requires extra components to handle network related issues like the routing of BATS packets, which are originally handled by the operating system. The application which uses a BATS code has to invoke the BATS protocol directly or has its packets forwarded via a local tunnel. The idea of local tunnel is that by setting the default gateway to the tunneling device, the traffic can be captured by an application which performs routing (by sending packets to a specific IP address which appears in the routing table) and also filters packets for BATS encoding. Therefore, we do not mix the packets that require the application of BATS codes and those that do not.

**Figure 24:**
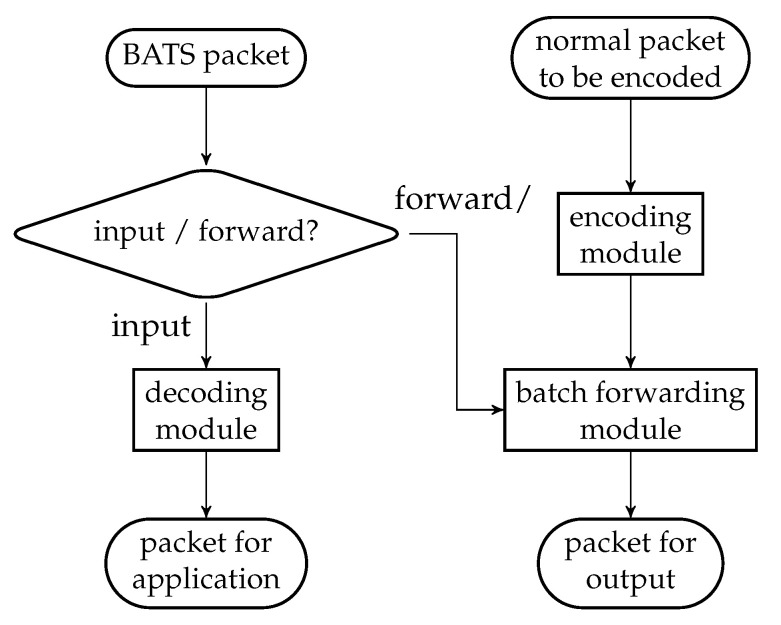
A packet flowchart for an application layer implementation which does not depend on packet filtering.

Although the current node is not the destination node, the destination IP address of any received BATS packet is the current node. In other words, the application which handles the incoming BATS packets has to distinguish the input traffic and the forward traffic. The packet flow is then simplified into the one illustrated in Figure 24.

### 5.2. Packet Design

We present the design of BATS packets in this subsection. We first discuss what kinds of information are necessary to be carried explicitly or implicitly by the BATS packets.

In order to decode the batches, the BATS decoder has to know how the raw packets in a batch are formed from the input packets, that is, it requires the knowledge of

1. the sizes and the irreducible polynomials of the base and recoding fields for the finite field arithmetic;
2. the length of the raw packets;
3. a unique identification to distinguish which batch the raw packets belong to;
4. the generator matrices of the batches;
5. the (ordered) index sets of the input packets involved to form the batches;
6. the batch transfer matrices; and
7. the size of the input data.

All the above information needs to be carried by the BATS packets. In addition, for the batch stream reconstruction in the ISMU, the BATS packets need to carry

8. the identification of the previous batch in the same batch stream.

#### 5.2.1. BATS-Pro1 Packets

We take the packet design of BATS-pro1 [41] as an example to illustrate how all the above information can be encapsulated in the BATS packet.

**Figure 25:**
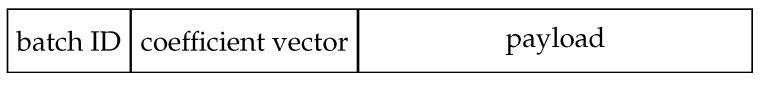
The three data fields of a BATS packet used by BATS-pro1.

The format of the BATS packets of BATS-pro1 is illustrated in Figure 25. There are only three data fields in a BATS packet, namely the *batch ID*, the *coefficient vector* and the *payload*. We can see that some necessary information is not explicitly carried by the BATS packet because we should make the overhead induced as small as possible to save bandwidth.

In BATS-pro1, it is assumed that the sizes and the irreducible polynomials of the finite fields are predefined at the encoder, recoders and decoder. This knowledge is necessary or otherwise we cannot interpret the received packets which are strings of bits or bytes. On the other hand, the protocol also assumes that

1. the batch size of all batches are fixed and known by the encoder, recoders and decoder; and
2. both the encoder and decoder know the size of the input data and the degree distribution.

The length of the raw packet, that is, the number of symbols in a raw packet, is known from the headers of the network stack. For example, the packet size including the headers (in bytes) is recorded in the IP header and UDP header, and so forth. From the packet size, the length of the raw packet can be determined. Thus we do not need to repeat this information in the BATS packet of BATS-pro1.

The unique identification for distinguishing the batches is the batch ID in the design. These batch IDs are also used at the recoders so that the network coding operations can be restricted to the BATS packets belonging to the same batch.

The columns of the batch transfer matrix of a batch are the coefficient vectors of the corresponding BATS packets of the batch, where these coefficient vectors are also used to record the network coding operations performed on the raw packets and indicate the linear independence among the raw packets (see Definition 2).

Recall that

1. the generator matrix is a totally random matrix over the base field;
2. the size of the index set is the degree of the batch which is sampled from the degree distribution; and
3. the elements in the index set are randomly chosen.

These randomness can be regenerated when we use the same seed with the same pseudorandom number generator (PRNG), for example, Mersenne Twister [88]. That is, when both the encoder and decoder use the same PRNG and use the same degree distribution, we only need to put the seed in the BATS packet. As the generator matrix and the index set of the input packets for the batch is independent of other batches, we can use a function of the batch ID as the seed. The simplest such function is the identity function, that is, the batch ID is the seed.

Note that the concepts of a batch stream and an ISMU do not exist in BATS-pro1, so we do not have the identification of the previous batch in the same batch stream included in the BATS packet. When there is no out-of-order packet, receiving a BATS packet having a new batch ID means that all the receivable packets of the previous batch are received. This was shown in the flowchart in Figure 2.

#### 5.2.2. General Packet Design

Except the three data fields used by BATS-pro1 and an extra field for the identification of the previous batch in the same batch stream, we need the BATS packets to carry more information in general to relax some assumptions made by BATS-pro1. In the general case, we may have

1. different field sizes used by different BATS codes;
2. different sizes of input data;
3. different batch sizes for different batches;
4. different degree distributions applied to suit the channel conditions of different paths;
5. more than one information source at a source node; and
6. more than one source node.

Although we can put the field sizes into the BATS packets, we can save some bits by restricting the choices of the finite fields as we are interested in a few of them which can be computed efficiently. For example, in most CPUs, we can extract the symbols of a finite field from a string of bits very efficiently when the field size is a power of 2, which is not the case if the field size is taken to be a power of other prime numbers. Also, we need extra bitwise operations to extract a symbol like those field sizes not in the form of $2^{2^{n}}$ when the basic unit of the CPU is the byte. That is, it is desirable to use field sizes equal to 2, 4, 16, 256, 65,536, and so forth. On the other hand, we may assume that the irreducible polynomials are predefined and agreed among the encoders and decoders so that we do not need to occupy more space in the BATS packets.

The size of the input data can affect the regeneration of the index sets of the input packets as it fixes the range when we sample the PRNG. Specifically, the decoder needs to know the total number of input packets, which can be deduced from the size of the input data and the length of the payload of a BATS packet (that is, the length of an input packet). Also, this information is useful when the size of the input data is not a multiple of the size (in bytes) of the input packets. In this case, some dummy symbols are appended to the input data, so we need to truncate these dummy symbols after decoding. A straightforward method to carry this information is to include a data field in the BATS packet which stores the size of the input data. Another way is to prepend the size of the input data to the input data itself before encoding and write the number of input packets as a data field in the BATS packets.

For the best performance of BATS codes, we need to optimize the degree distributions according to the channel conditions. On the other hand, even when the batch size is fixed, we may have more than one degree distribution for a single BATS encoder, for example, Reference [89]. So, we need to put the information about the degree distribution used by the batch, for example, the distribution itself, the maximum batch size and maximum batch degree, in at least some of the BATS packets in that batch. Note that the length of a coefficient vector is related to the batch size, so we also need to include the batch size in the BATS packet when it is not a constant among all the encoders.

The batch ID is only for the identification of batches within the same information source. When there are more than one information source at a source node, we need to extend the identification to include some information about the information source. For example, we can

- use a counter or random number generator to assign a number to different information sources; or
- use a random source port number if the BATS protocol is carried by UDP; or
- use a cryptographic hash function on the input data of the information source.

The first method is similar to the idea of port number in TCP and UDP, while the last one is similar to the idea of deduplication in file systems. We call the batch ID with the aforementioned extension the *extended batch ID*.

**Definition** **10.** *An extended batch ID is a unique identification of a batch among different information sources at the same source node.*

When there is more than one source node, the extended batch ID is not sufficient to distinguish the traffic from different source nodes, for example, two source nodes can transmit identical input data with the same extended batch ID. However in general, there should have unique identifications to distinguish the two source nodes like MAC addresses, IP addresses, and so forth. We can use the same information an NAT device uses to distinguish the source nodes. For example, if we implement the BATS protocol over UDP, then the pairs of IP addresses and port numbers of the source and destination nodes are used for this purpose. This idea is also valid after this information is modified by an NAT device as our goal is to distinguish the traffic from different source nodes.

**Figure 26:**
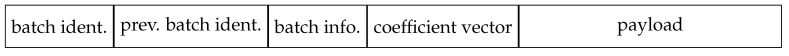
The data fields of a general BATS packet. The words ident., prev. and info. are the abbreviations of identification, previous and information respectively.

As a brief summary, a BATS packet has the following data fields as shown in Figure 26:

- the identification of the batch the BATS packet belongs to, where part of this identification can be used as a seed for a PRNG to regenerate the necessary parameters at the decoder;
- the identification of the previous batch in the same batch stream, which can be used to distinguish different batches of different information sources from different source nodes;
- the batch specific information, like the field sizes, the batch size, the size of the input data or the number of input packets, the degree distributions, and so forth;
- the coefficient vector for the payload, which records the network coding operations; and
- the payload, which is the body of the BATS data, that is, a linearly transformed raw packet.

To ensure data integrity, we can further introduce a data field for a checksum, for example, the Internet checksum which is also applied in the UDP and IP headers. Other than a checksum, we can also use a message authentication code or a digital signature for unforgeability.

On the other hand, if we allow mixing more than one batch for recoding like the operations in FUN codes [50,51], we need to further extend the idea of extended batch ID so that it can include the identifications of the batches involved. We can also include some other side information like the rank of the batch at the current node in the BATS packet. Nevertheless, these application-wise extensions still follow the design paradigm in this paper.

#### 5.2.3. Initialization of Coefficient Vectors

When the raw packets of a batch are freshly produced by the BATS encoder, they are regarded as being linearly independent of each other. As we have coefficient vectors in the BATS packets, we can use them to record this linearly independence relation. However, the BATS decoder has to know the initial coefficient vectors in order to be able to recover the input data correctly.

Let *M* be the batch size of a batch and $e_{1},e_{2},\ldots ,e_{M}$ be the column coefficient vectors of the batch. Each coefficient vector has *M* symbols over the recoding field where each symbol corresponds to one of the raw packets in the batch. Then these raw packets are linearly independent of each other if and only if the M×M matrix $\left(e_{1},e_{2},\ldots ,e_{M}\right)$ is full rank.

One simple such full rank matrix is an M×M identity matrix over the recoding field. By using the identity matrix, we can see directly from the coefficient vectors how the raw packets are mixed by linear combinations.

#### 5.2.4. First Packet of a Batch Stream and Dummy Packets

We have to define the identification of the previous batch in the same batch stream when such a batch does not exist. We also need to make sure that this identification does not collide with those identifications of other existing batch streams. One simple solution is to define the previous batch to be the same as the current batch, which obviously will not occur in the existing batch streams.

On the other hand, we need a design on the dummy packets so that they can be identified. The only necessary information a dummy packet has to carry is the batch identification which informs the ISMU that the corresponding batch stream is broken. So, we can design a dummy packet as a BATS packet which only has the data field for the batch identification. The absence of the remaining data fields can be detected by reading the size of the BATS packet.

## 6. Application Examples

In this section, we will discuss some examples to demonstrate how to use the BATS protocol.

### 6.1. Relay Network

**Figure 27:**
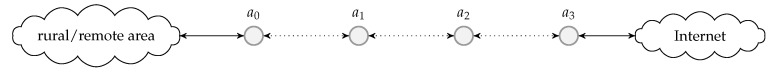
The network topology of an example of providing Internet access to a rural/remote area.

One main reason for applying BATS codes is to carry network traffic over multiple wireless links. We have presented a straightforward example in Section 4.5. Figure 20 in Section 4.5 can be interpreted as a simple example to provide Internet access to rural and remote areas. That is, we can regard node $a_{0}$ as a gateway of the rural/remote area network, where all the traffic from the area to the Internet must pass through node $a_{0}$. The links from node $a_{0}$ to node $a_{1}$, from node $a_{1}$ to node $a_{2}$ and from node $a_{2}$ to node $a_{3}$ are all wireless links, where node $a_{3}$ can access the Internet via a wired connection. The network topology for this case is shown in Figure 27, where the dotted edges represent the wireless links.

As the encoding and decoding modules are deployed at nodes $a_{0}$ and $a_{3}$, the traffic passing through the wired links are not BATS packets. That is, the BATS packets are decoded before a network address translation (NAT) is performed if there is any.

In order to prevent a single point of failure, the rural/remote area network and the Internet should connect to more than one gateway. On the other hand, there should be a backup path for the wireless network so that when one or more nodes fail to operate, the rural/remote area and the Internet are still connected. If possible, we can introduce more wireless nodes to form a wireless mesh network so that there can be more than one path for the connection. In case we cannot introduce more wireless nodes, then we should allow the establishment of a wireless link that hops over the field node. For example, if node $a_{1}$ in Figure 27 fails to operate, then a wireless link connecting nodes $a_{0}$ and $a_{2}$ is established. After the routing is updated, the connection is resumed. However, we need to implement a mechanism to restore the original wireless connection when the failed node works again. This is independent of the BATS protocol so it is outside the scope of this paper.

### 6.2. Internet Sharing

One application of BATS codes is to provide WiFi hotspots at locations where the access points (APs) have no direct access to the Internet. For example, these APs can be installed on the RSUs in a VANET [11] or on the trees in a country park.

The scenario is similar to the case in the last subsection. The wireless nodes this time are the RSUs along a street or the trees along a hiking trail. Only some if not all of the wireless nodes are APs. At least one of the wireless node has access to a wired network, which is a gateway to the Internet. It may not be feasible to apply decoders at such a gateway node for all the BATS traffic passing through it as it is a device installed on-site, that is, it is likely that the device has a lower computational power. We can introduce another node with higher computational power for decoding which is not on-site but connecting to those gateway nodes by wireline.

**Figure 28:**
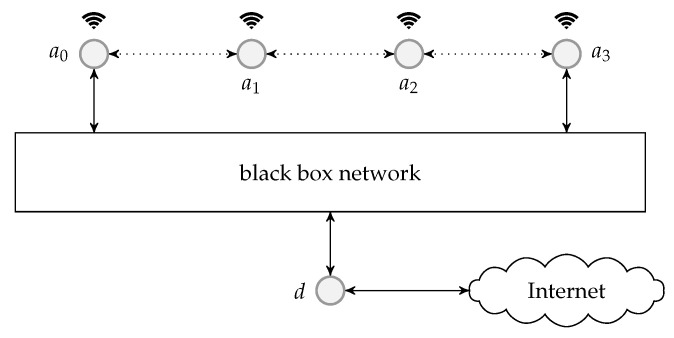
An example network topology for Internet sharing.

Figure 28 illustrates an example of the network topology. The nodes $a_{i}$ where i=0,1,2,3 are APs. We call them the *on-site nodes*. In the example, only nodes $a_{0}$ and $a_{3}$ have wired connection to node *d* through a black box network. The black box network is an arbitrary network which we may not have permission to configure. Node *d* has Internet access and has multiple encoding and decoding modules deployed.

In the above setting, BATS packets have to pass through the black box network, although the nodes in the black box network do not need to perform recoding. We prefer to choose a “shorter path” to route a packet from an AP to node *d*. There are various approaches to decide the route if we only consider the number of hops in the distance measure. For example, if we use the transport layer implementation, then we can use a network bridge among node *d* and the on-site nodes (It requires the enabling of the 4-address mode to use the 4-address frame format in the IEEE 802.11 frame header). A Layer 2 Tunneling Protocol (L2TP) can be applied to form tunnels between nodes $a_{0}$ and *d* and between nodes $a_{3}$ and *d* so that the black box network is transparent to the network bridge. The Spanning Tree Protocol (STP) of the network bridge with node *d* being a root bridge can ensure that the number of hops between an on-site node and the root bridge is minimal. If we use the application layer implementation which depends on packet filtering, then we can apply existing routing protocols like Open Shortest Path First (OSPF) and Routing Information Protocol (RIP) at least on the on-site nodes.

Note that it may not be the best strategy to choose the path with the smallest number of hops as we should also take the wireless signal conditions into account. There exist some advanced routing protocols which are designed for this purpose and suitable to be applied on wireless mesh networks, for example, Better Approach To Mobile Adhoc Networking (B.A.T.M.A.N.) and Babel.

**Figure 29:**
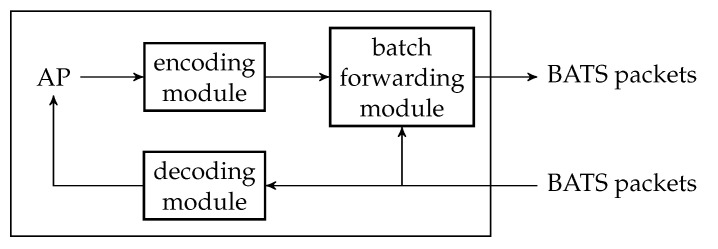
The assembly of the modules of BATS protocol for Internet sharing at an on-site node without wired connection.

Now we consider the assembly of the modules of the BATS protocol. An encoding module, a decoding module and a batch forwarding module are deployed at node *d* and all the on-site nodes. The basic relation between the modules at an on-site node without wired connection is shown in Figure 29. An incoming BATS packet is passed to

- the batch forwarding module if the on-site node is not the destination of the packet; and/or
- the decoding module if the destination of the packet is the on-site node.

In other words, a BATS packet will be passed to both the batch forwarding module and the decoding module if

- the packet is a broadcast packet; or
- the packet is a multicast packet and the on-site node is one of the multicast targets.

At an on-site node with wired connection, the packets from the AP can pass through the wired network directly without applying BATS codes. Therefore, the traffic from the AP will not go into the encoding module unless the wired connection is disconnected.

**Figure 30:**
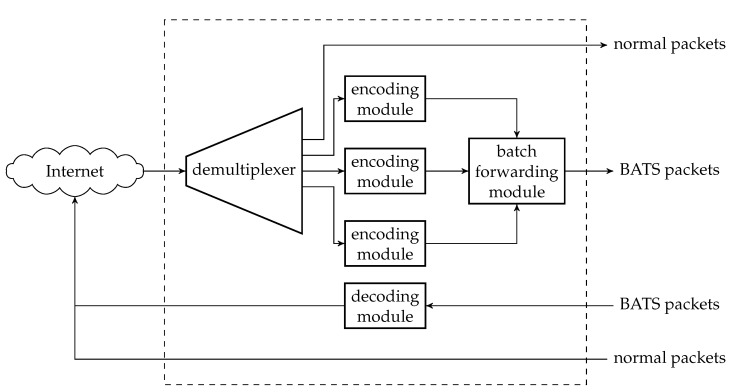
An example of the assembly of the modules of BATS protocol for Internet sharing at node *d* which has access to the Internet directly.

Node *d* is a gateway for the on-site nodes to access the Internet. It decodes the BATS packets before they reach the Internet and encodes the Internet traffic where the destinations are the on-site nodes without wired connection. Figure 30 illustrates an example of the assembly of the modules of BATS protocol at node *d*. The incoming Internet packet is passed to a demultiplexer and classified according to the destination. There are two cases:

1. if the destination is an on-site node which has a direct wired connection to node *d*, then the packet is forwarded to that on-site node without applying BATS codes; otherwise
2. the traffic is passed to the corresponding encoding module for that node to apply BATS codes.

On the other side, there are two cases for the incoming packet from the on-site nodes:

1. if the packet is a BATS packet, that is, it is from an on-site node without direct wired connection to node *d*, then it is passed to a decoding module which may have multiple BATS decoders in it; otherwise
2. the packet is forwarded to the Internet directly.

In order to prevent a single point of failure and achieve high availability, we need to have other gateway nodes except node *d* which can connect to the Internet. At the same time, we need some on-site nodes which can reach these gateway nodes directly via the black box network. If the black box network also plays the role of a load balancer, the gateway nodes may need to forward BATS packets to each other for decoding.

For example, suppose the policy distributes the packets to two gateway nodes $d_{1}$ and $d_{2}$. We need to feed the BATS packets of the input data of the same information source to the same decoding module so that the BATS decoder can receive enough batches for recovering the input data. So, a connection between the nodes $d_{1}$ and $d_{2}$ is established to exchange the BATS packets when the corresponding BATS decoder is not located at the node. We can predefine a scheme to decide which node applies the BATS decoder of the input data of the information source, for example, one of the node is responsible for a predefined subset of the on-site nodes while the other one is responsible for the remaining ones.

### 6.3. Wireless Mesh Network

The network topologies in the examples we shown in the previous two subsections are simple. In a more general setting, we may have a wireless mesh network where the network nodes can join and leave the network at any moment. Also, there may be multiple available paths between a pair of source and destination nodes. The network topology is not static in a wireless mesh network. One such example is the VANET [11].

BATS packets of a batch can be routed through different paths, that is, the BATS packets can be distributed to different intermediate nodes. Those intermediate nodes can only perform recoding on the received packets. If the BATS packets of a batch reach the node which applies the BATS decoder to that batch from multiple links, then we can feed the packets to the decoding module directly. However, when the BATS packets of a batch reach an intermediate node from multiple links, we need to merge these BATS packets for recoding.

**Figure 31:**
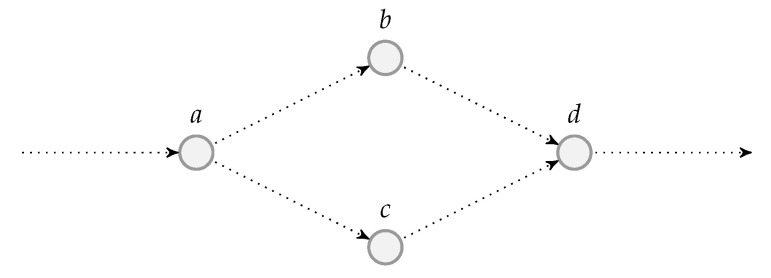
An example of a part of a wireless mesh network.

Take the network topology shown in Figure 31 as an example. All the nodes shown in the figure are intermediate nodes. Suppose the BATS packets of a batch at node *a* are distributed to nodes *b* and *c*. Recoding of this batch is performed independently at nodes *b* and *c*. The recoded packets from these two nodes reach the same node *d*. At node *d*, the BATS packets of this batch should be merged for recoding.

By using identifications like the MAC addresses or the IP addresses of nodes *b* and *c*, we can distinguish the received BATS packets from the two nodes so that we can reconstruct different batch streams in the ISMU at node *d* even if the previous batch identification in the BATS packets are the same. Recall that the batch identification in a BATS packet is a unique identification to distinguish different batches of different information sources from different source nodes, so the batch buffer at node *d* knows that the received packets are from the same batch and thus can merge them in the buffer. Although there may be a significant time difference between the reception of those BATS packets from nodes *b* and *c*, this issue can be handled by the mechanism for handling out-of-order packets in the batch forwarding module.

### 6.4. Network Address Translation for Captured Traffic

**Figure 32:**
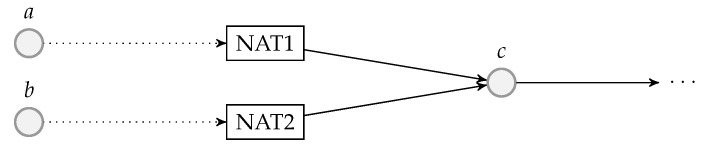
A simple network where nodes *a* and *b* have the same IP address.

The traditional NAT devices allow the destination node to transmit packets back to the source node even when there are nodes having the same IP address in different subnets. For example, suppose the source nodes *a* and *b* in Figure 32 have the same IP address. The two NAT devices in the figure have distinct IP addresses as seen by node *c*. That is, when a BATS packet transmitted by node *a* reaches node *c*, the source IP address is changed to the IP address of NAT1 as seen by node *c*. Another piece of information is changed by the NAT, for example, the source port number for a UDP packet. In other words, there is enough information to distinguish the traffic from different source nodes so that we will not mix up the batches from different source nodes. Together with the extended batch ID, we can feed the batches to the right BATS decoder to recover the input data.

Note that an NAT device has to reassemble the IP fragments before it applies the translation because it needs to recalculate the Internet checksums. We can reduce the delay induced by the reassembly if the length of a BATS packet together with the other headers is no more than the maximum transmission unit (MTU), that is, no IP fragmentation is required to transmit the BATS packet.

There is an issue when the input data is formed by a captured traffic although the BATS decoder recovers the input data correctly. Specifically, the source IP addresses (and source port numbers if there are any) of the captured packets are not modified by the NAT. We need to apply another layer of NAT which maps the pairs of source information in a BATS packet and a captured packet into a new set of source information. We can see the reason from the example below.

**Figure 33:**
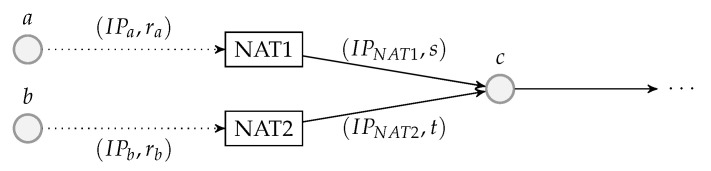
An example of the source IP address and source port number pairs of the BATS packets on each link, where $IP_{a}=IP_{b}$.

Suppose nodes *a* and *b* in Figure 32, which have the same IP address, transmit two UDP packets each. Let the source IP address and source port number pairs of the UDP packets be $\left(IP_{a},p_{a}\right)$, $\left(IP_{a},q_{a}\right)$, $\left(IP_{b},p_{b}\right)$ and $\left(IP_{b},q_{b}\right)$ respectively, where the subscripts indicate the source nodes. Further suppose the port numbers of the packets from the two nodes collide, that is, we have $IP_{a}=IP_{b}$, $p_{a}=p_{b}$ and $q_{a}=q_{b}$. These packets are captured and encoded by BATS codes. BATS decoders are deployed at node *c* so that the captured packets are recovered before forwarding. For demonstration purpose, suppose the BATS protocol is implemented over UDP. Then, node *a* transmits a sequence of BATS packets with source IP address and source port number pair $\left(IP_{a},r_{a}\right)$. Similarly for node *b*, we have the pair $\left(IP_{b},r_{b}\right)$. The NAT device to which node *a* connects maps the pair $\left(IP_{a},r_{a}\right)$ to the pair $\left(IP_{\mathrm{NAT}1},s\right)$, and the one to which node *b* connects maps the pair $\left(IP_{b},r_{b}\right)$ to the pair $\left(IP_{\mathrm{NAT}2},t\right)$. We have $IP_{\mathrm{NAT}1}\neq IP_{\mathrm{NAT}2}$ so that node *c* can distinguish the two NAT devices. Figure 33 illustrates the source IP address and source port number pairs of the BATS packets on each link.

After decoding at node *c*, the source information of the packets are summarized in Table 4. We must substitute the IP addresses of the recovered captured packets as those IP addresses are not reachable from node *c*. Recall that $\left(IP_{a},p_{a}\right)=\left(IP_{b},p_{b}\right)$ and $\left(IP_{a},q_{a}\right)=\left(IP_{b},q_{b}\right)$ by our construction, but we need to be able to tell that they are from different source nodes. That is why we also need to use the source information of the BATS packets as part of the mapping for the NAT.

The above issue can be prevented if the BATS packets do not pass through NAT devices directly, for example, a tunnel is established so that the BATS packets only travel within the same subnet.

## 7. Performance Evaluation

In this section, we use a line network with artificial burst packet loss as an example to illustrate the performance (including throughput and latency) of the recoding and interleaving schemes proposed in this paper by making comparison with certain benchmark protocols.

### 7.1. Suggestions on the Choice of Parameters

If an input packet is too long, the coded packets need to be separated into multiple frames for transmission by the data link layer, which may increase the chance of packet loss. On the other hand, if an input packet is too small, the overall overhead induced by the headers and the coefficient vectors of the packets can be significant. We now give a simple example to illustrate the design considerations. In the WiFi and cellular network standards, we can set the length of the payload of the input packets to be 1024 bytes. Then this payload together with the necessary headers can be transmitted as one frame at the data link layer, without exceeding the maximum transmission unit (MTU) whose default value is 1500 bytes. Packets of this length, together with headers, can be transmitted usually using one data link layer frame, and are long enough so that the overhead is relatively small.

Let *M* be the batch size. It was shown in Reference [3] that the throughput gap between M=16 and 32 is much smaller than the gap between M=8 and 16. In other words, M=16 is a suitable choice where the throughput is satisfactory and the overhead is acceptable. We suggest to use M=16 in practical systems if the computational cost is manageable at the recoders.

We tend to use a reasonably large recoding field instead of a binary recoding field—is a trade-off between throughput and computational cost. It was suggested and shown in References [3,44] that a recoding field GF(256) has a performance close to a recoding field which size tends to infinity. Also, it is easy to handle GF(256) operations in modern digital devices as 1 byte is enough to represent all the symbols in GF(256) so that the operations are simply bytes operations. Therefore, unless the computational power of the recoders is severely limited, we prefer GF(256) to GF(2) as the recoding field.

Before performing the evaluation, we first introduce the latency definition, the burst loss model and the interleaver depth selection.

### 7.2. Throughput

The theoretical upper bound on the end-to-end *normalized throughput* of a BATS code is the expected value of the rank of the batches arriving at the destination node divided by the batch size [3], while a BATS code can achieve a normalized throughput very close to this upper bound. That is, we define

$$\mathrm{normalized}\;\mathrm{throughput}=\frac{\mathrm{expected}\;\mathrm{value}\;\mathrm{of}\;\mathrm{the}\;\mathrm{rank}\;\mathrm{of}\;\mathrm{the}\;\mathrm{received}\;\mathrm{batches}}{\mathrm{batch}\;\mathrm{size}}.$$

This performance metric was also considered in works such as References [30,44,79].

We will verify in our simulation that the throughput of a BATS code can be degraded by burst loss, and an interleaver can boost the throughput significantly.

### 7.3. Latency Ratio

In a traditional packet network, the latency is the time between the moment a packet is transmitted by the source node and the moment the destination node receives the packet. When we apply BATS codes, the packets transmitted from an intermediate node are new packets generated by recoding, that is, the packets received by the node are being mixed in order to generate recoded packets. For example, a linearly dependent packet of a batch arriving at an intermediate node will be absorbed by the node so that the content of this packet will not be directly transmitted to the next node. However, the content of this packet is encoded into all of the recoded packets of the same batch which are not systematically recoded. We need to extend the definition of latency so that it can quantify the performance of BATS codes.

We first consider a traditional packet network. When we regard a packet transmitted by the source node as a file, the reception of the packet at the destination node means that the file is being received successfully. In other words, we can define latency as the time between the moment the source node starts transmitting a file and the moment the destination node successfully recovers the file. This definition can be applied to the networks using BATS codes.

In the paper, we adopt the above definition of latency. We define the *latency ratio* by normalizing the latency by the number of input packets of the file, that is,

$$\mathrm{latency}\;\mathrm{ratio}=\frac{\mathrm{latency}}{\mathrm{number}\;\mathrm{of}\;\mathrm{input}\;\mathrm{packets}}.$$

This ratio is the average latency per input packet.

Note that when the number of input packets is 1, the definition of latency ratio collide with the one of latency. However, a BATS code with only one input packet degenerates into a repetition code, as the next node only has to receive one of the coded packets in one of the batches in order to fully recover the file. That is, when we apply systematic recoding, the latency of the BATS code in this scenario is the same as the latency of link-by-link retransmission scheme with instantaneous and reliable feedback. Although we do not have feedback for the BATS code, we can achieve this optimal latency regardless of the interleaver depth.

### 7.4. Simplified Gilbert Model

We use a simplified *Gilbert model* [80] to model the link condition for burst packet loss. A more elaborate model proposed in Reference [81] which generalizes the Gilbert model is known as the *Gilbert-Elliott model*. Although the Gilbert-Elliott model has a better ability to capture the burst loss pattern than the simplified Gilbert model, the simplicity of the latter model allows simple analysis and estimation of bursty links, for example, Reference [90]. We leave a description of the Gilbert-Elliott model to Appendix B.

**Figure 34:**
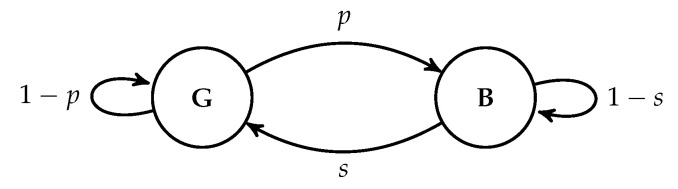
A simplified Gilbert model.

A simplified Gilbert model is a two-state Markov chain illustrated in Figure 34. When a packet is being transmitted through the link, we first update the state of the Markov chain. If the state is the good state **G**, then the next node receives the packet successfully. Otherwise, that is, the state is the bad state **B**, the packet is being dropped by the link.

Once we fall into the bad state, a burst of packet loss begins. The number of time slots before we escape from the bad state is the length of the burst error. That is, the length of the burst error follows a geometric distribution. The mean of the distribution is 1/s, which is also known as the average burst error length (ABEL) [91]. On the other hand, the average packet loss rate $p_{E}$ is the stationary state probability of the bad state. The stationary distribution $\left(\pi_{G},\pi_{B}\right)$ of the Markov chain can be determined as

$$\pi_{G}=\frac{s}{p+s}\;\mathrm{and}\;\pi_{B}=\frac{p}{p+s}.$$

Note that we can model independent packet loss using the simplified Gilbert model by $p=p_{E}$ and $s=1-p_{E}$. This implies that independent packet loss is a special case of burst packet loss where ABEL is $1/(1-p_{E})$.

When we know the average packet loss rate $p_{E}$ and the average burst error length ABEL, the transition probabilities can be determined as

$$p=\frac{p_{E}}{(1-p_{E})\mathrm{ABEL}}\;\mathrm{and}\;s=\frac{1}{\mathrm{ABEL}},\;\mathrm{when}\;p_{E}>0.$$

Otherwise, when $p_{E}=0$, we set p=0 and s=1.

### 7.5. Interleaver Depth

Let *L* be the interleaver depth. We want to select a suitable *L* which can cover most of the bursts so that the burst can be spread efficiently. Recall that the burst error length in the simplified Gilbert model follows a geometric distribution. Therefore, the sum of the first *L* probability masses of the geometric distribution is the portion of all bursts covered by the interleaver. If we want the interleaver to cover at least η portion of all bursts, then we need an *L* satisfying

(3) $$\sum_{i=0}^{L-1}s{\left(1-s\right)}^{i}=1-{\left(1-s\right)}^{L}\geq \eta .$$

**Figure 35:**
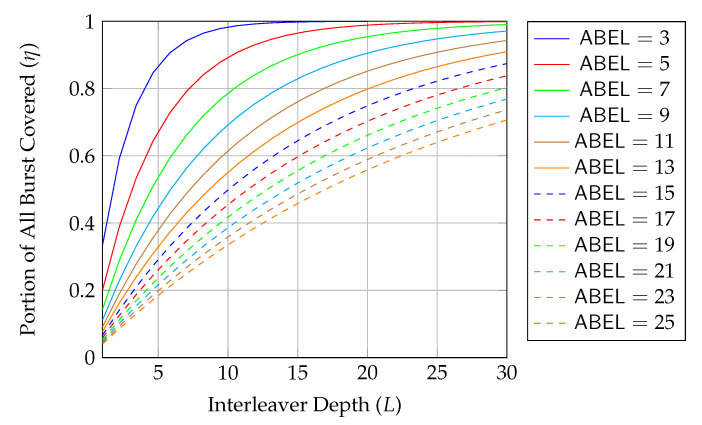
The relation between the interleaver depth and the portion of all bursts covered for different average burst error length.

Figure 35 is a plot of the left hand side of (3) on different ABEL where s=1/ABEL, which illustrates the relation between the interleaver depth and the portion of all bursts covered. We can see that when ABEL is not large, a small interleaver depth can already provide a good coverage of the bursts.

There is no burst when there is no packet loss and there is no way to cover a non-zero portion of all bursts when the length of any burst is infinity, so we only consider 0<s<1 in this subsection. Note that log(1−s)<0, so we have $L\geq \log_{1-s}\left(1-\eta \right)$ by rearranging (3). That is, the smallest integer *L* in order to cover at least η portion of all bursts is

$$L=\lceil \log_{1-s}\left(1-\eta \right)\rceil .$$

Recall that ABEL is the average length of the burst. If we choose an *L* which equals the ABEL, we can in fact cover at least 63.21% of all the bursts. This number can be derived as follows. By substituting s=1/L into (3), we have $1-{\left(1-1/L\right)}^{L}\geq \eta$. Note that $1-{\left(1-1/L\right)}^{L}$ is strictly decreasing for L≥1, and it tends to 1−1/e when L→∞, where *e* is Euler’s number. Therefore, we have η≥1−1/e≈63.21%.

### 7.6. Simulation

In the following evaluation, we consider a line network of 10 hops, that is, there are totally 11 nodes including the source node. There is no feedback available in this network for BATS codes. The channel condition of each link is modelled by a simplified Gilbert model. For demonstration purposes, we use the same channel condition where $p_{E}=20\%$ and ABEL=5 for all the links. That is, the transition probabilities are p=0.05 and s=0.2.

We assume the same constant transmission rate at all the nodes. We divide the timeline into multiple time slots where each time slot has the same duration. At most one packet can be transmitted per time slot. For simplicity, we assume a synchronized timeline for all the nodes, that is, all the nodes transmit a packet (if there is any) at the same time. It can be achieved in practice by using different channels of the wireless medium. A packet transmitted at time slot *t* will be received by the next node at time slot t+1 if it is not dropped.

We use GF(256) as both the base field and the recoding field. For each set of simulation using BATS codes, we use a constant batch size for all the batches and a constant interleaver depth at all the links. We try both batch sizes M=8 and 16 in the evaluation. For the interleaver depth, we try both L=4 and 8, which correspond to η=59.04% and 83.22% respectively. The source node transmits *M* packets per batch. We start recoding of a batch after the node has received all the receivable packets of this batch. If baseline recoding is used as the recoding scheme, then the recoder generates *M* packets per batch if at least one packet of the batch is received. For adaptive recoding, we set $t_{\mathrm{avg}}=M$ so that we have a fair comparison. As the receiving state of a packet of a batch is determined after updating the Markov chain *L* times, we use the *L*-th power of the transition matrix of the simplified Gilbert model in the calculation of the expected rank functions.

We apply the protocol proposed in this paper in four different configurations as follows:

1. All nodes use adaptive recoding and transmit causally recoded packets when there is an idle time slot;
2. All nodes use baseline recoding and transmit causally recoded packets when there is an idle time slot;
3. All nodes use adaptive recoding but not causal recoding;
4. All nodes use baseline recoding but not causal recoding.

Also, we apply systematic recoding at all the nodes for all the four configurations. The timeout for identifying a broken stream is ML time slots.

As a comparison, we also evaluate the throughput and latency ratio of other protocols listed below.

- Interleaved BATS-pro1: a benchmark of using BATS codes.
- End-to-end retransmission with instantaneous and reliable feedback: a lower bound for the protocols which use end-to-end-retransmission. This bound is also true for fountain codes without feedback.
- Link-by-link retransmission with instantaneous and reliable feedback: an ideal capacity achieving scheme which consumes high bandwidth for feedback.

We do not apply interleavers to the retransmission schemes so that their latency is minimized. The lack of interleavers will not affect the throughput of the other protocols as stated in Table 1.

For TCP, the feedback, that is, the acknowledgement (ACK) with the correct sequence number, has to travel back to the source node so it is likely to be lost in multi-hop wireless networks. TCP would restart the slow start procedure when no ACK is received after a timeout so that the transmission rate would be kept very low. In the simulation, our end-to-end retransmission setting prevents the above scenario to be happened. So, our result is the best case TCP can obtain when the maximum transmission rate is one packet per time slot, where this limited rate is a fairness concern to other protocols to be compared which do not have rate control algorithms.

On the other hand, note that the link-by-link retransmission scheme is capacity achieving so we will see that its throughput is the highest and its latency is the smallest. However, it requires feedback while the BATS protocols do not.

In the simulation, the number of input packets is 16,000. We record the throughput and the latency ratio at each hop, which can be viewed as the throughput and latency ratio when this hop is a destination node.

#### 7.6.1. Throughput

**Figure 36:**
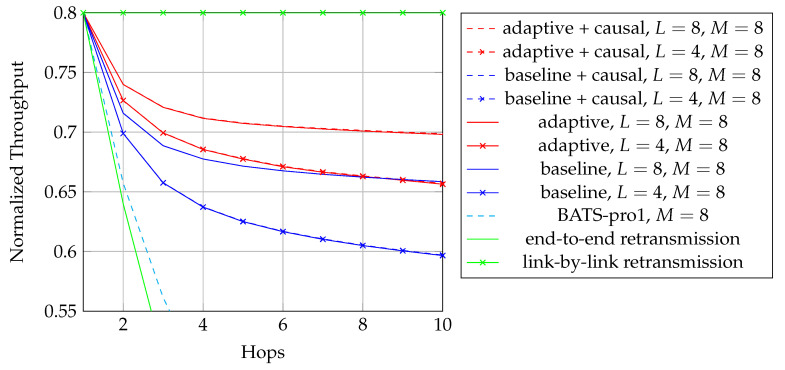
The normalized throughput of different protocols when the batch size is 8.

**Figure 37:**
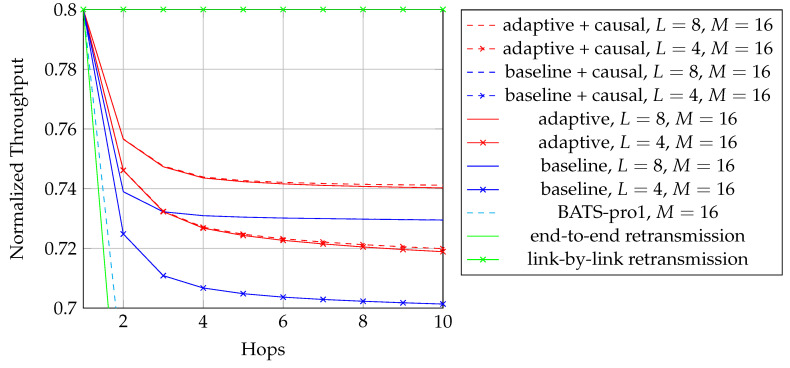
The normalized throughput of different protocols when the batch size is 16.

**Figure 38:**
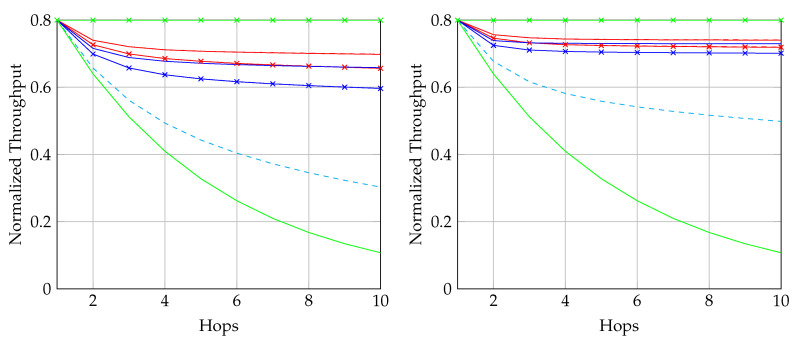
The left and the right plots are the full plots of those shown in Figure 36 and Figure 37 respectively.

Figure 36 and Figure 37 show the throughput of different protocols when the batch sizes *M* is 8 and 16 respectively. As interleaved BATS-pro1 uses baseline recoding, the throughput of interleaved BATS-pro1 is the same as our proposed protocol with baseline recoding. So, we do not plot interleaved BATS-pro1 in the figures. The full plots of Figure 36 and Figure 37 are drawn in Figure 38.

We have the following observations:

1. A larger batch size can result in a higher normalized throughput.
2. The throughput of BATS codes can be boosted dramatically with the help of interleavers.
3. When the interleaver depth is larger, the throughput is also higher.
4. Adaptive recoding has a higher throughput than baseline recoding when the packet loss pattern is independent [30,44], but now we can see that this is also true in burst packet loss pattern.
5. Transmitting a causally recoded packet when there is an idle time slot has little impact on the throughput. The difference is noticeable only when the batch size and the number of hops become large.

#### 7.6.2. Latency Ratio

**Figure 39:**
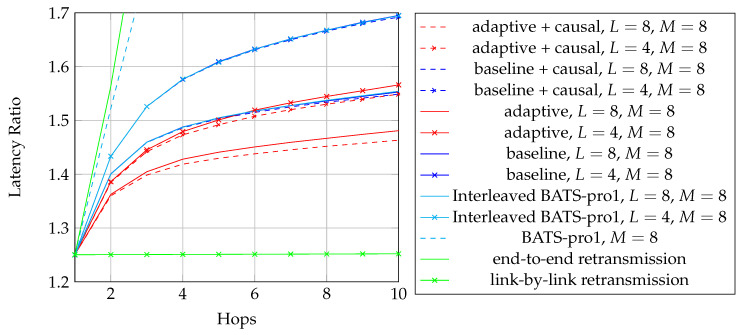
The latency ratios of different protocols when the batch size is 8.

**Figure 40:**
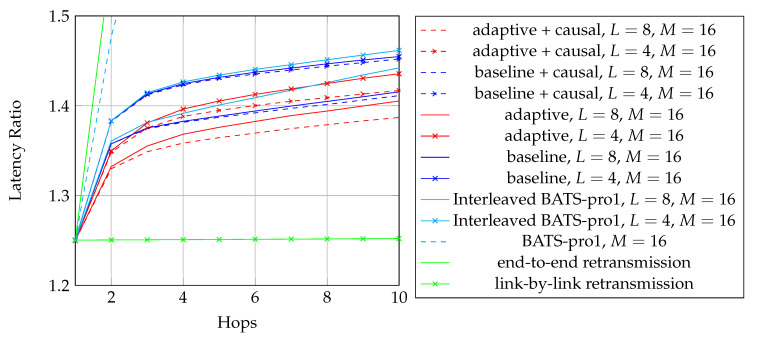
The latency ratios of different protocols when the batch size is 16.

**Figure 41:**
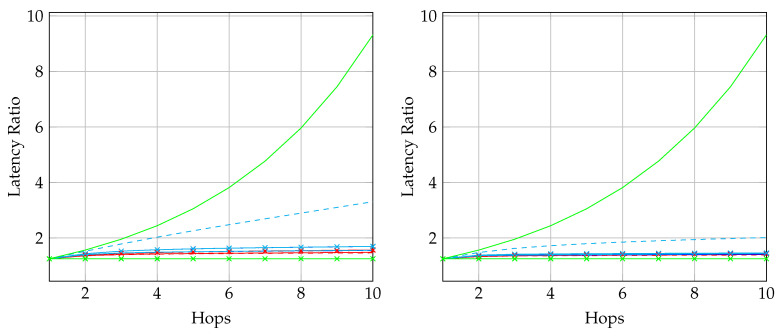
The left and the right plots are the full plots of those shown in Figure 39 and Figure 40 respectively.

Figure 39 and Figure 40 shows the latency ratios of different protocols when the batch sizes *M* is 8 and 16 respectively. The full plots of Figure 39 and Figure 40 are drawn in Figure 41 to illustrate how bad the end-to-end transmission scheme and BATS codes without interleavers are.

We have the following observations:

1. The latency ratio of the end-to-end retransmission scheme is rocketing when the number of hops increases. We can see that it is not practical to use end-to-end retransmission schemes in multi-hop wireless networks.
2. Although comparing with the end-to-end retransmission scheme, BATS codes without interleavers have a much better performance, the latency ratio is still increasing too fast in multi-hop wireless networks.
3. The latency ratio when using L=8 is lower than that when using L=4. We can further compare with the one without interleavers, which has a significantly worse performance. This verifies the detrimental performance implication due to burst loss on BATS codes as suggested in Reference [41].
4. The transmission of causally recoded packets when there is an idle time slot can lower the latency ratio. This is because these causally recoded packets have a chance to secure some ranks of the batches at the next node.
5. Adaptive recoding achieves a lower latency ratio than baseline recoding, as the throughput of adaptive recoding is higher than the one of baseline recoding.
6. BATS-pro1 has a higher latency ratio than its corresponding scheme using the interleaver proposed in this paper with systematic recoding. This is an expected result as we have to transmit fewer (non-systematically) recoded packets after all receivable packets of a batch are received. The gap between the latency ratios is more significant when batch size is larger. This suggests that systematic recoding has a greater benefit when the batch size is larger.
7. The latency ratio is larger when the batch size is smaller, as BATS codes have a lower throughput for smaller batch size. Also, we can see that when there is no interleaver, a larger batch size has a better resistance against performance degradation due to burst loss than a smaller batch size.

## 8. Conclusions

BATS codes are a class of practical network coding schemes which can asymptotically achieve rates very close to the capacity of a packet network with packet loss. The adoption of BATS codes require a BATS protocol which is different from the traditional end-to-end protocol because we need to apply recoding at at least some of the intermediate network nodes to gain advantage over store-and-forward.

There are different recoding schemes to explore the potential of BATS codes in different directions. On the other hand, the strategy for combating burst packet loss is a crucial part which can affect the throughput of BATS codes significantly. In this paper, we proposed a protocol design paradigm for BATS codes which can support arbitrary recoding schemes and burst loss handling techniques.

The core idea of the design is to use the facts that recoding is an intra-batch operation while the handling of burst loss is an inter-batch operation. It is realized by performing the inter-batch operations among different batch streams, where each batch stream is a concatenation of individual batches applying the intra-batch operations. The inter-batch operations are rewound at the receiver side by simulating the batch streams at the transmitter side. A discussion on which kinds of information have to be carried by the BATS packets is also included.

## 9. Patents

The foundation of the protocol design in this paper can be found in the U.S. patent 10,425,192 invented by the authors of this paper, which was granted on 24 September 2019 [86].

## Figures and Tables

**Table 1:** Protocol Independent Comparisons.

	Feedback for Reliability	Asymptotic Throughput (Independent Loss)	Storage Cost at Intermediate Node	Coding Coefficient Overhead	Performance Degradation due to Burst Loss
fountain codes	no	${\left(1-p_{E}\right)}^{\ell }$	O(T)	O(1)	no
RLNC	no	$1-p_{E}$	O(KT)	O(K)	no
disjoint BNC	link-by-link	$\frac{\left(1-p_{E}\right)\left(T-M\right)}{T}$	$\Omega (\sqrt{p_{E}KT})$	O(M)	no
BATS codes	no	$\frac{\left(1-p_{E}\right)\left(T-M\right)}{T}$	O(MT)	O(M)	yes
end-to-end retransmission	end-to-end	${\left(1-p_{E}\right)}^{\ell }$	O(T)	no	no
link-by-link retransmission	link-by-link	$1-p_{E}$	$\Omega (\sqrt{p_{E}KT})$	no	no

**Table 2:** Organization of the Paper.

BATS codes	Section 2: Brief introduction Section 3: Recoding schemes Appendix A, Appendix B and Appendix C: Some analysis of recoding
BATS protocol	Section 4: Module design Section 5: Packet design
Demonstration	Section 6: Examples of applying the BATS protocol Section 7: Simulations of the BATS protocol
Conclusion	Section 8: Conclusions of the paper

**Table 3:** The bijective mapping managed by the ISMU where the corresponding batch streams reconstruction is shown in Figure 10.

Batch Stream	Batch
2	$c_{4}$
4	$a_{4}$
5	$d_{2}$

**Table 4:** The source information of the packets after decoding at node *c*.

BATS Packet	Captured Packet
$\left(IP_{NAT1},s\right)$	$\left(IP_{a},p_{a}\right)$
$\left(IP_{NAT1},s\right)$	$\left(IP_{a},q_{a}\right)$
$\left(IP_{NAT2},t\right)$	$\left(IP_{b},p_{b}\right)$
$\left(IP_{NAT2},t\right)$	$\left(IP_{b},q_{b}\right)$

**Figure A1:**
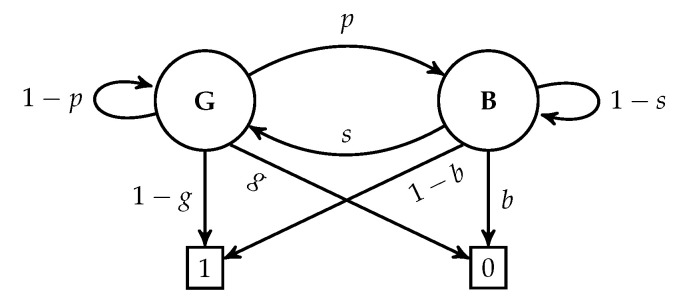
The Gilbert-Elliott model for burst channels.

**Figure A2:**
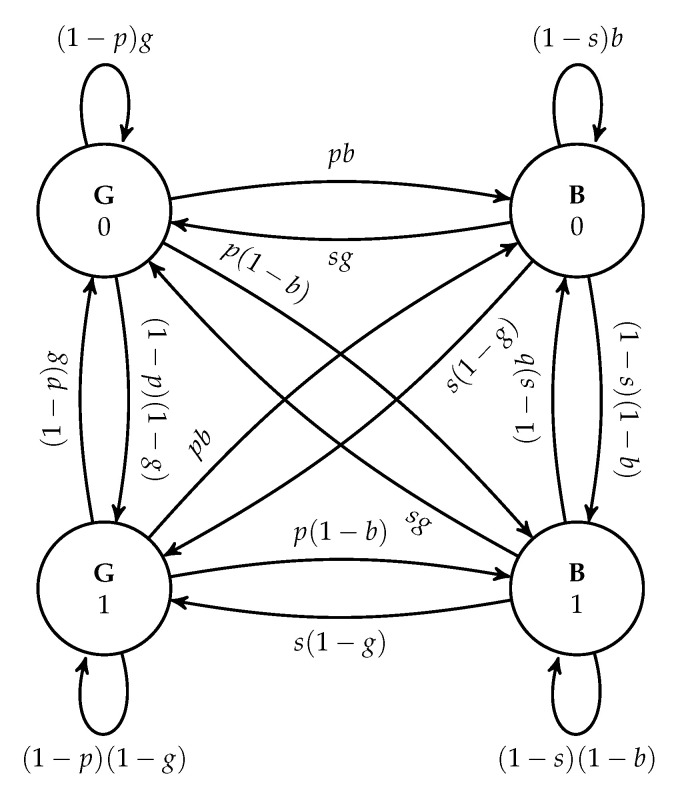
The Gilbert-Elliott model shown in Figure A1 after expanding the hidden Markov structure.

**Figure A3:**
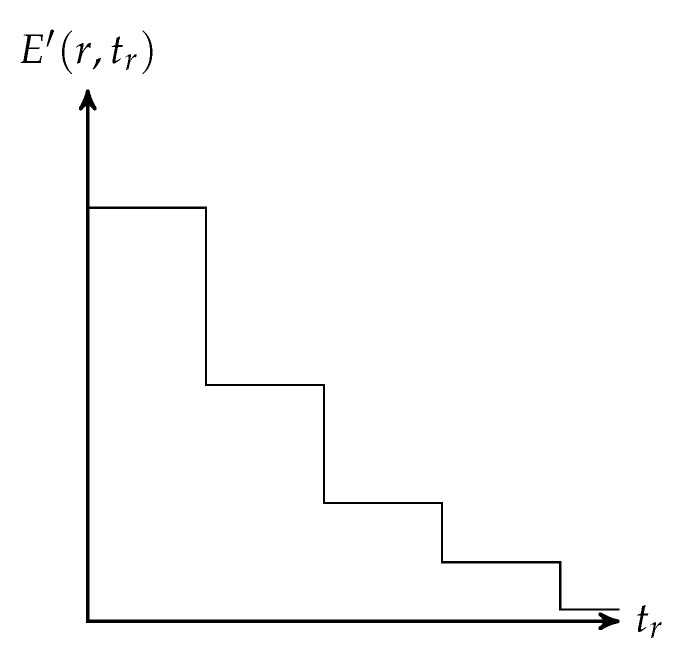
A plot of $E^{\prime }\left(r,t_{r}\right)$, which is the superdifferential of $E(r,t_{r})$ with respect to $t_{r}$.

**Figure A4:**
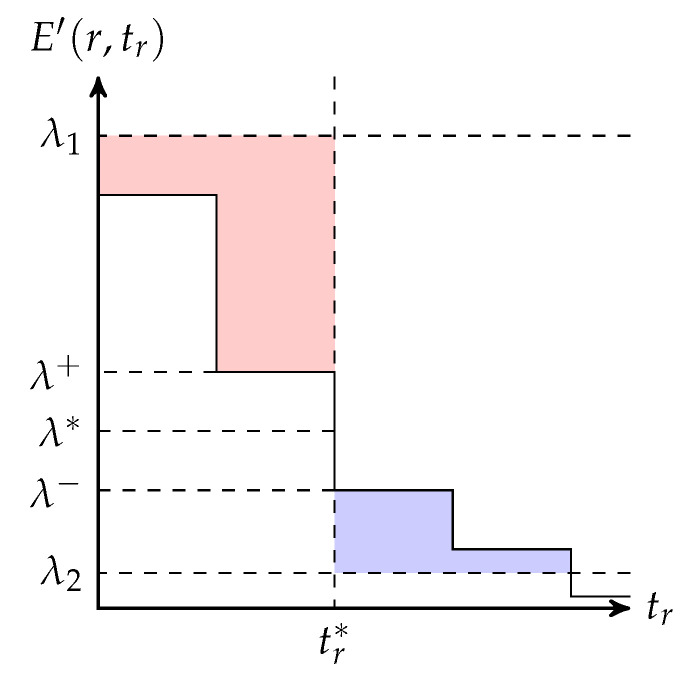
This figure illustrates the integral $\int_{\lambda^{*}}^{\lambda }\left(t_{r}^{*}-t_{r}\left(\sigma \right)\right)\;d\sigma$. When the optimal $t_{r}^{*}$ is an integer, the optimal λ is a value in a specific subinterval of the interval $\left[\lambda^{-},\lambda^{+}\right]$ and the value of the integral is 0. Those values $\lambda_{1}>\lambda^{+}$ and $\lambda_{2}<\lambda^{-}$ are not optimal. The areas of the red and blue regions are the values of the integral when λ equals $\lambda_{1}$ and $\lambda_{2}$ respectively.

## Abbreviations

The following abbreviations are used in this manuscript:

BATS codes	Batched sparse codes
IoT	Internet of things
VANET	Vehicular ad hoc network
RSU	Road side unit
RLNC	Random linear network codes/random linear network coding
BNC	Batched network coding
LT codes	Luby transform codes
FUN codes	Joint fountain coding and network codes
CPU	Central processing unit
SIMD	Single instruction, multiple data
SSSE3	Supplemental Streaming SIMD Extensions 3
BP	Belief propagation
i.i.d.	Independent and identically distributed
NP	Non-deterministic polynomial-time
ident.	Identification
prev.	Previous
info.	Information
NUM	Network utility maximization
ISMU	Input stream management unit
OSMU	Output stream management unit
NIC	Network interface card
MAC	Medium access control
IP	Internet protocol
ICMP	Internet control message protocol
UDP	User datagram protocol
TCP	Transmission control protocol
ATCP	Ad hoc TCP
FASP	Fast adaptive and secure protocol
mDNS	Multicast domain name system
NTP	Network time protocol
L2TP	Layer 2 tunneling protocol
STP	Spanning tree protocol
OSPF	Open shortest path first
RIP	Routing information protocol
B.A.T.M.A.N.	Better approach to mobile adhoc networking
NAT	Network address translator/network address translation
PRNG	Pseudorandom number generator
ABEL	Average burst error length
AP	Access point
MTU	Maximum transmission unit
ACK	Acknowledgement

## Appendix A. Proper Recoding

*Proper recoding* [5] is one of the ways to define reasonable behaviours of a recoder. Based on the observations in Reference [44], proper recoding is defined by three conditions to be satisfied. Consider two batches, where the first batch has rank $r_{1}$ and the second batch has rank $r_{2}$. Let $t_{1}$ and $t_{2}$ be the number of recoded packets to be generated for the first and second batches respectively.

**Definition** **A1.**
*A recoder is proper if and only if it satisfies all the following conditions:*

1. *If $r_{1}=r_{2}$ and $t_{1}=t_{2}$, then both batches have the same rank distribution at the next network node;*
2. *If $r_{1}>r_{2}$ and $t_{1}=t_{2}$, then the first batch potentially has a higher rank at the next network node; and*
3. *If $r_{1}=r_{2}$ and $t_{1}>t_{2}$, then the first batch potentially has a higher rank at the next network node.*

The first condition suggests that a recoder should be consistent with the rank of the recoded packets when the rank of the received packets and the number of recoded packets are the same, that is, a recoder is not biased towards any batch.

The second condition implies that a batch with higher rank should result in a higher rank at the next node than a batch with a lower rank. It is reasonable because more linearly independent packets are embedded in the recoded packets of the first batch.

The last condition asserts that if we generate more recoded packets, we should have a chance to sustain a higher rank at the next node. It is because a newly generated recoded packet has a chance to be received and be linearly independent of all the previously received packets of the same batch at the next node.

There is no specification on the formal definition of “potentially has a higher rank”, but it should have the *transitivity property*. For example, the definition used in References [5,64] is the *first-order stochastic dominance* [74] between the rank distributions where transitivity holds. When transitivity holds, we have the following lemma.

**Lemma** **A1.**
*Assume the recoder is proper. If $r_{1}>r_{2}$ and $t_{1}>t_{2}$, then the first batch potentially has a higher rank at the next network node.*

**Proof** We consider a third batch, which has rank $r_{3}$ and generated $t_{3}$ recoded packets such that $r_{1}>r_{3}=r_{2}$ and $t_{1}=t_{3}>t_{2}$. The second and third conditions of proper recoding assert that the first batch potentially has a higher rank than the third batch at the next node, and the third batch potentially has a higher rank than the second batch at the next node. The proof is done by applying the transitivity property. □

We can compare with the discussion on the properties of the expected rank functions in Appendix B to see that the expected rank functions for “fair” channels reassemble proper recoding. That is, we can define the first batch to be potentially having a higher rank than the second batch at the next node if the expected rank of the first batch is no less than the one of the second batch. Note that the definition of proper recoding by first-order stochastic dominance implies the above expected rank property [74], but the converse is not true in general, and so the definition of proper recoding by expected rank functions is actually a weaker version.

## Appendix B. Expected Rank Functions

Denote by E(r,t) the expected rank function with a batch of rank *r* and *t* transmitted recoded packets. We must have E(r,0)=0≤E(r,t)≤r for all non-negative integers *r* and *t*, because

- the next node must receive no packet if no packet is transmitted, that is, the expected rank is 0; and
- the linear combinations of *r* independent vectors cannot result in more than *r* independent vectors.

In general, suppose we model the channel of an outgoing link by a stochastic process $\left\{X_{t}\right\}$, where $X_{t}$ are S-valued random variables. We define a function f:S→{0,1} to indicate whether a packet is received or not. Then we can write $Z_{t}=f\circ X_{t}$ for all *t*, where $\left\{Z_{t}\right\}$ is a sequence of binary random variables forming a stochastic process. Let $Z_{t}=1$ if the *t*-th packet is received at the next node, and $Z_{t}=0$ otherwise. Let $Z_{1}$ be the random variable for the first transmitted packet of a batch. Then, the expected rank is expressed by

$$E\left(r,t\right)=\sum_{i=0}^{t}\mathrm{Pr}\left(\sum_{j=1}^{t}Z_{j}=i\right)\tilde{E}\left(r,i\right),$$

where $\tilde{E}\left(r,i\right)$ is the expected value of the rank of a batch at the next node when the batch has rank *r* at the current node and *i* packets received at the next node.

If RLNC is used to generate all the recoded packets, we have

$$\tilde{E}\left(r,i\right)=\sum_{j=0}^{\min\{i,r\}}j\zeta_{j}^{i,r},$$

where $\zeta_{j}^{i,r}$ is the probability (in terms of recoding field size $q^{\prime }$) that a batch of rank *r* at the current node with *i* received packets at the next node has rank *j* at the next node. The exact expression of $\zeta_{j}^{i,r}$ can be found in Reference [3], which is

$$\zeta_{j}^{i,r}=\frac{\zeta_{j}^{i}\zeta_{j}^{r}}{\zeta_{j}^{j}{\left(q^{\prime }\right)}^{\left(i-j)(r-j\right)}},$$

where

$$\zeta_{j}^{m}=\left\{\begin{array}{cc} \left(1-{\left(q^{\prime }\right)}^{-m}\right)\left(1-{\left(q^{\prime }\right)}^{-m+1}\right)\ldots \left(1-{\left(q^{\prime }\right)}^{-m+j-1}\right) & \mathrm{if}\;j>0,\\ 1 & \mathrm{if}\;j=0. \end{array}\right.$$

The value of $\zeta_{j}^{i,r}$ is either very close to 1 or 0 when the recoding field size $q^{\prime }$ is large enough. When $q^{\prime }$ tends to infinity, we have

$$\lim_{q^{\prime }\to \infty }\zeta_{j}^{i,r}=\left\{\begin{array}{cc} 1 & \mathrm{if}\;j=\min\{r,i\},\\ 0 & \mathrm{otherwise}. \end{array}\right.$$

For a batch of rank *r*, the above limit means that a newly received packet at the next node is linearly independent almost surely of all the previously received packets, unless the next node has already received *r* packets. Practical implementations in References [41,44] suggest that the above limit can give a close approximation when the recoding field size is $2^{8}$. In this case, we have

$$E\left(r,t\right)=\sum_{i=0}^{t}\mathrm{Pr}\left(\sum_{j=1}^{t}Z_{j}=i\right)\min\left\{i,r\right\}.$$

It was shown in Reference [82] that when RLNC is used to generate all the recoded packets and the channel model $\left\{X_{t}\right\}$ follows a stationary stochastic process, then the expected rank function E(r,t) is monotonic increasing and concave with respect to *t* regardless of the recoding field size. Note that if $\left\{X_{t}\right\}$ itself is a sequence of binary random variables and *f* is the identity function, then we have $Z_{t}=X_{t}$ for all *t*, that is, the stationarity of $\left\{X_{t}\right\}$ is inherited by $\left\{Z_{t}\right\}$. This inheritance also holds in general, which is stated in the following theorem.

**Theorem** **A1.**
*If $\left\{X_{t}\right\}$ is a stationary stochastic process, then so is $\left\{f\circ X_{t}\right\}$.*

**Proof** Let $f^{-1}\left(b\right)\subseteq S$ be the preimage of *b*, where b=0,1. For any finite set of indices $\left\{t_{1},\ldots t_{k}\right\}$, any integer τ, and any $\left(b_{1},\ldots ,b_{k}\right)\in {\left\{0,1\right\}}^{k}$, we have

(A1) $$\begin{array}{c} \mathrm{Pr}(Z_{t_{1}+\tau }=b_{1},\ldots ,Z_{t_{k}+\tau }=b_{k})\\ \;=\mathrm{Pr}(X_{t_{1}+\tau }\in f^{-1}\left(b_{1}\right),\ldots ,X_{t_{k}+\tau }\in f^{-1}\left(b_{k}\right))\\ \;=\mathrm{Pr}(X_{t_{1}}\in f^{-1}\left(b_{1}\right),\ldots ,X_{t_{k}}\in f^{-1}\left(b_{k}\right))\\ \;=\mathrm{Pr}(Z_{t_{1}}=b_{1},\ldots ,Z_{t_{k}}=b_{k}), \end{array}$$

where (A1) holds because $\left\{X_{t}\right\}$ is stationary. As the joint distribution is the same after an arbitrary time shift, we know that $\left\{Z_{t}\right\}$ is also stationary. □

A more refined discussion in Reference [92] showed that the concavity of E(r,t) with respect with *t* is strict at t=c≥1 for all r>0 when

- $q^{\prime }$ is finite and $\mathrm{Pr}(Z_{1}=1,Z_{c+1}=1)\neq 0$; or
- $q^{\prime }\to \infty$ and $\mathrm{Pr}(Z_{1}=1,Z_{c+1}=1,\sum_{i=2}^{c}Z_{i}=r-1)\neq 0$.

Also, Reference [92] showed that E(r+1,t+1)−E(r+1,t)≥E(r,t+1)−E(r,t) where the inequality is strict if

- $q^{\prime }$ is finite and $\mathrm{Pr}(Z_{t+1}=1)\neq 0$; or
- $q^{\prime }\to \infty$ and $\mathrm{Pr}(Z_{t+1}=1,\sum_{i=1}^{t}Z_{i}=r)\neq 0$,

which implies that E(r+1,t)≥E(r,t) where the inequality is strict if t>0 and the above conditions are satisfied. When the channel is “fair”, we must have a chance to receive any subset of the transmitted packets from the previous node, so that the above four conditions must be satisfied.

It is trivial that the channel model $\left\{X_{t}\right\}$ follows a stationary stochastic process when the packet loss events are independent. For dependent packet loss events, the channel can be modeled by a multi-state stochastic process. A higher number of states can model the channel more accurately [93,94,95]. However, it may still not be accurate enough to model the number of consecutively received packets [96].

A widely used burst channel model, the *Gilbert-Elliott model* [80,81] as shown in Figure A1, has only two states. More precisely, it is a hidden Markov chain model with two states and two possible observations. The two states **G** and **B** in the Gilbert-Elliott model correspond to the good and bad states respectively. In each state, there are two possible observations 1 and 0, which decide whatever a packet is received or not.

To see why the observations of the Gilbert-Elliott model follow a stationary stochastic process, we can expand the model into a four-state Markov chain as shown in Figure A2. Then, we have S={(G,0),(G,1),(B,0),(B,1))}.

Note that the Markov chain shown in Figure A2 is time-homogeneous, that is, stationary. By applying Theorem A1, we know that the observations of the Gilbert-Elliott model follow a stationary stochastic process, and thus the corresponding expected rank function is concave. We can apply similar arguments to burst channel models with more states.

## Appendix C. Global Asymptotic Stability of Dual-Based Solver for Adaptive Recoding Schemes

We consider a dual-based solver which applies a subgradient searching method to the primal problem (2). By Slater’s condition, the strong duality holds for (2).

The Lagrangian is

$$L\left(t,\lambda \right)=\sum_{r=0}^{M}h_{r}E\left(r,t_{r}\right)-\lambda \left(\sum_{r=0}^{M}h_{r}t_{r}-t_{\mathrm{avg}}\right),$$

where λ≥0 is the Lagrange multiplier and $t=(t_{0},t_{1},\ldots ,t_{M})$. Then, we can write down the dual formulation of (2), which is

$$\min_{\lambda \geq 0}\underset{t_{r}\geq 0,\forall r}{\sup}L\left(t,\lambda \right).$$

Rearranging the terms, we have

$$\min_{\lambda \geq 0}\left(\sum_{r=0}^{M}\underset{t_{r}\geq 0}{\sup}\left(h_{r}\left(E\left(r,t_{r}\right)-\lambda t_{r}\right)\right)+\lambda t_{\mathrm{avg}}\right),$$

which becomes a form suitable for applying the dual decomposition [97].

A dual-based solver performs the following two steps iteratively. Let $\lambda^{\left(k\right)}$ be the Lagrange multiplier in the *k*-th iteration.

1. Given $\lambda^{\left(k\right)}$, find a value of $t_{r}$ for each *r* satisfying
  (A2) $$\underset{t_{r}\geq 0}{\sup}\left(E\left(r,t_{r}\right)-\lambda^{\left(k\right)}t_{r}\right);$$
2. From the $t_{r}$ obtained in the first step, adjust the Lagrange multiplier by a subgradient searching method, that is,
  (A3) $$\lambda^{\left(k+1\right)}=\max\left\{0,\lambda^{\left(k\right)}+\alpha^{\left(k\right)}\left(\sum_{r=1}^{M}h_{r}t_{r}-t_{\mathrm{avg}}\right)\right\},$$
  where $\alpha^{\left(k\right)}>0$ is the step size in the *k*-th iteration.

One can to choose $\alpha^{\left(k\right)}$ arbitrarily as long as the subgradient search converges, for example, a diminishing step size or a small constant. We can also simulate binary search with a specially designed sequence of step sizes. However, the global asymptotic stability analysis does not depend on the selected sequence of step sizes.

Recall that the expected rank function $E(r,t_{r})$ is formed by linear interpolations and it is non-decreasing and concave with respect to $t_{r}$. We can define the superdifferential of $E(r,t_{r})$ which is an interval of all positive supergradients at $t_{r}$. We denote the superdifferential with respect to $t_{r}$ by $E^{\prime }\left(r,t_{r}\right)$. Figure A3 illustrates a plot of $E^{\prime }\left(r,t_{r}\right)$. The horizontal lines correspond to the line segments formed by linear interpolations. The vertical lines only occur when $t_{r}$ is an integer, that is, the original discrete points before the linear interpolations, which correspond to the non-differentiable points of $E(r,t_{r})$.

Note that in (A2), a λ can correspond to more than one value of $t_{r}$ for each *r* due to the non-differentiability. Denote all those values of $t_{r}$ given a λ by the mapping $t_{r}\left(\lambda \right)$. According to Reference [82], the plot can also be interpreted as a plot of Lagrange multiplier λ verse $t_{r}$ for the batches of rank *r*. That is, we can evaluate the mapping $t_{r}\left(\lambda \right)$ easily from Figure A3. When a λ (in the y-axis) corresponds to a single value of $t_{r}$ (in the x-axis), the mapping $t_{r}\left(\lambda \right)$ gives a single integer point. Otherwise, the mapping $t_{r}\left(\lambda \right)$ gives a single interval. The exact form of the mapping can be found in Reference [82]. In other words, an optimal Lagrange multiplier can correspond to multiple feasible primal solutions, but we can then apply the tuning schemes proposed in Reference [82] to obtain an optimal primal solution.

**Theorem** **A2.**
*The dual-based solver is globally asymptotically stable.*

**Proof** We first define a function and show that it is a Lyapunov function candidate. Then we apply the Lyapunov global asymptotic stability theorem [98] to conclude the stability directly. Define a shifted Lyapunov function

$$V\left(\lambda \right)=\left(t_{\mathrm{avg}}-\sum_{r=1}^{M}h_{r}t_{r}^{*}\right)\lambda +\sum_{r=1}^{M}h_{r}\int_{\lambda^{*}}^{\lambda }\left(t_{r}^{*}-t_{r}\left(\sigma \right)\right)\;d\sigma ,$$

where $t_{r}^{*}$ and $\lambda^{*}$ are one of the optimal primal solutions and one of the optimal dual solutions respectively. To prove that V(λ) is a Lyapunov function candidate which can achieve global asymptotic stability, we need to show that

1. for V(λ)≥0, the equality holds if and only if λ is optimal;
2. for $\dot{V}\left(\lambda \right)\leq 0$, the equality holds if and only if λ is optimal; and
3. the radial unboundedness condition holds, that is, $\lim_{\lambda \to \infty }V\left(\lambda \right)=\infty$.

We must achieve the constraint $\sum_{r=1}^{M}h_{r}t_{r}^{*}=t_{\mathrm{avg}}$ as $t_{r}^{*}$ is optimal, so $t_{\mathrm{avg}}-\sum_{r=1}^{M}h_{r}t_{r}^{*}$ is always equal to 0. As $h_{r}\geq 0$, we only need to consider the term $\int_{\lambda^{*}}^{\lambda }\left(t_{r}^{*}-t_{r}\left(\sigma \right)\right)\;d\sigma$ for the first condition. If $\lambda >\lambda^{*}$, we have $t_{r}\left(\sigma \right)\leq t_{r}^{*}$. If $\lambda <\lambda^{*}$, we have $t_{r}\left(\sigma \right)\geq t_{r}^{*}$. Therefore, we have V(λ)≥0 for all λ≥0. We now consider two cases:

1. If $t_{r}^{*}$ is not an integer, then there is a unique optimal solution $\lambda^{*}$. So the integral $\int_{\lambda^{*}}^{\lambda }\left(t_{r}^{*}-t_{r}\left(\sigma \right)\right)\;d\sigma =0$ if and only if λ is optimal.
2. If $t_{r}^{*}$ is an integer, then there exists an interval $\left[\lambda^{-},\lambda^{+}\right]$ such that, if $t_{r}\left(\sigma \right)=t_{r}^{*}$, then σ falls in that interval. That is, $\int_{\lambda^{*}}^{\lambda }\left(t_{r}^{*}-t_{r}\left(\sigma \right)\right)\;d\sigma =0$ if and only if $\lambda \in [\lambda^{-},\lambda^{+}]$.

The condition when the integral $\int_{\lambda^{*}}^{\lambda }\left(t_{r}^{*}-t_{r}\left(\sigma \right)\right)\;d\sigma$ equals 0 is shown in Figure A4. Note that the optimal λ across all *r* is a subinterval $\Lambda \subseteq [\lambda^{-},\lambda^{+}]$. That is, there exists an *r* such that $\int_{\lambda^{*}}^{\lambda }\left(t_{r}^{*}-t_{r}\left(\sigma \right)\right)\;d\sigma \neq 0$ if $\lambda \in [\lambda^{-},\lambda^{+}]\setminus \Lambda \neq Ø$. The above arguments show that V(λ)=0 if and only if λ is optimal. For the second condition, we first evaluate $\dot{V}\left(\lambda \right)$.

$$\begin{array}{cc} \dot{V}\left(\lambda \right) & =\left(t_{\mathrm{avg}}-\sum_{r=1}^{M}h_{r}t_{r}^{*}\right)\dot{\lambda }+\sum_{r=1}^{M}h_{r}\left(t_{r}^{*}-t_{r}\left(\lambda \right)\right)\dot{\lambda }\\ \; & =\left(t_{\mathrm{avg}}-\sum_{r=1}^{M}h_{r}t_{r}\left(\lambda \right)\right)\dot{\lambda }\\ \; & \leq 0, \end{array}$$

where $\dot{\lambda }$ is the change in λ induced by (A3). We have $\dot{V}\left(\lambda \right)=0$ if and only if a $t_{r}\left(\lambda \right)$ is found such that $\sum_{r=1}^{M}h_{r}t_{r}\left(\lambda \right)=t_{\mathrm{avg}}$, that is, an optimal λ is found. The third condition can be verified trivially from Figure A4. When λ tends to infinity, the area of the red region also tends to infinity. As all the three conditions are satisfied, we conclude that the dual-based solver is globally asymptotically stable by the Lyapunov global asymptotic stability theorem. □
